# An Essential Farnesylated Kinesin in *Trypanosoma brucei*

**DOI:** 10.1371/journal.pone.0026508

**Published:** 2011-11-02

**Authors:** Erin J. Engelson, Frederick S. Buckner, Wesley C. Van Voorhis

**Affiliations:** 1 Department of Medicine, Division of Allergy and Infectious Diseases, University of Washington, Seattle, Washington, United States of America; 2 Molecular and Cellular Biology Program, University of Washington, Seattle, Washington, United States of America; 3 Department of Global Health, University of Washington, Seattle, Washington, United States of America; 4 Department of Microbiology, University of Washington, Seattle, Washington, United States of America; University of Texas-Houston Medical School, United States of America

## Abstract

Kinesins are a family of motor proteins conserved throughout eukaryotes. In our present study we characterize a novel kinesin, Kinesin^CaaX^, orthologs of which are only found in the kinetoplastids and not other eukaryotes. Kinesin^CaaX^ has the CVIM amino acids at the C-terminus, and CVIM was previously shown to be an ideal signal for protein farnesylation in *T. brucei*. In this study we show Kinesin^CaaX^ is farnesylated using radiolabeling studies and that farnesylation is dependent on the CVIM motif. Using RNA interference, we show Kinesin^CaaX^ is essential for *T. brucei* proliferation. Additionally RNAi Kinesin^CaaX^ depleted *T. brucei* are 4 fold more sensitive to the protein farneysltransferase (PFT) inhibitor LN-59, suggesting that Kinesin^CaaX^ is a target of PFT inhibitors' action to block proliferation of *T. brucei*. Using tetracycline-induced exogenous tagged Kinesin^CaaX^ and Kinesin^CVIMdeletion^ (non-farnesylated Kinesin) expression lines in *T. brucei*, we demonstrate Kinesin^CaaX^ is farnesylated in *T. brucei* cells and this farnesylation has functional effects. In cells expressing a CaaX-deleted version of Kinesin, the localization is more diffuse which suggests correct localization depends on farnesylation. Through our investigation of cell cycle, nucleus and kinetoplast quantitation and immunofluorescence assays an important role is suggested for Kinesin^CaaX^ in the separation of nuclei and kinetoplasts during and after they have been replicated. Taken together, our work suggests Kinesin^CaaX^ is a target of PFT inhibition of *T. brucei* cell proliferation and Kinesin^CaaX^ functions through both the motor and farnesyl groups.

## Introduction

*Trypanosoma brucei* species are the causative agents of Human African Trypanosomiasis (HAT) or African sleeping sickness in humans and the wasting disease, nagana, in cattle. There were 9878 new HAT cases reported to the WHO in 2009 [1], notably the first decline below 10,000 reported cases since 1960 due in part to increased national sleeping sickness control programs [1] and disease mapping [2]. However, as resources are limited in many parts of rural Africa and surveillance in many areas is not yet routine, many cases go unreported. The WHO estimates from 30,000 to 70,000 new cases of HAT occur per year [3]. *Trypanosoma* species can also infect livestock including goats, sheep, pigs, donkeys and cattle [4]. This has an impact economically as many parts of Africa are unable to raise livestock for consumption and sale due to this parasite [4].

Currently no vaccines are effective at preventing *Trypanosoma brucei* infections. Existing medical therapies do exist, however many are toxic, require long treatment regimens and are difficult to administer [3]. Drug resistance is also a concern [5]–[8] and new drugs are urgently needed.

In our search for possible drug targets against protozoan parasites we have characterized the enzymes responsible for protein prenylation [9]–[19]. Prenylation is the posttranslational modification of proteins by the covalent addition of the isoprenyl lipid farnesyl or geranylgeranyl [20], [21]. In farnesylation, the fifteen carbon farnesyl group from farnesyl pyrophosphate is added to the C of the CaaX motif, a cysteine-containing four amino acid residue motif at the C-terminus of some proteins. The aa represent two aliphatic residues and the X represents amino acids including serine, methionine, alanine, threonine or glutamine [20]. Geranylgeranylation refers to the addition of a twenty-carbon geranylgeranyl group to the CaaX motif where X is commonly a leucine or phenylalanine. Prenylation modifications create a hydrophobic C-terminus that allows the protein to interact with the cell membrane, membrane-bound organelles, other cellular proteins and hydrophobic surfaces. Addition of the farnesyl or geranylgeranyl groups is mediated in mammalian cells by three heterodimeric enzymes: protein farneysltransferase (PFT), protein geranylgeranyltransferase type I (PGGT-I) and protein geranylgeranyltransferase type II (PGGT-II) [20], [21].

Previously our work has investigated *T. brucei* PFT (TB-PFT) enzyme as a potential drug target for developing new drugs against *T. brucei* [9]–[16]. TB-PFT covalently-links a farnesyl group from farnesyl-pyrophosphate to the cysteine on the CaaX motif. In mammals, both PFT and PGGT-I share the α-subunit, but bioinformatic studies suggest that *T. brucei* lacks a gene encoding the β-subunit of PGGT-I and biochemical studies suggest *T. brucei* lacks PGGT-I activity [14]. Our group has shown that PFT inhibitors inhibit prenylation of *T. brucei* proteins and inhibit *T. brucei* growth [10]–[11]. We have also demonstrated that *T. brucei* parasites are more sensitive to PFT inhibitors than *T. cruzi* parasites [18]. We have identified a *T. cruzi* gene encoding the β-subunit homolog of PGGT-I, Gene DB# Tc00.1047053508817.150 [18]. PGGT-I has been shown to act on many of the same substrates that PFT normally prenylates [20] and *T. cruzi* PGGT-I may provide a redundant function for PFT and thus explain why *T. cruzi* is less sensitive to PFT inhibitors than *T. brucei*. Taken together, these data suggest TB-PFT is a valid target for drug development for HAT therapy.

Conventionally the X amino acids in CaaX motifs modified by mammalian PFT include serine, methionine, alanine and glutamine. In contrast, our work has shown that while TB-PFT readily farnesylates CaaX targets containing a methionine or glutamine in the X position, it does not readily modify CaaX targets with serine, threonine, alanine or cysteine in the X position [12]. We have also identified inhibitors that are ten-fold more potent against TB-PFT than mammalian PFT [11]. This restricted preference to TB-PFT and not the mammalian PFT is desirable for drug design targeted to kill *T. brucei* in humans but not affect the human form of the enzyme. However, of the PFT inhibitors that had sufficient potency against TB-PFT, none had desirable *in vivo* pharmacokinetics [15]. Therefore we began the study of the preferred CaaX containing proteins that have the methionine in the X position to investigate proteins downstream of TB-PFT in order to identify new drug targets.

We have shown the CVIM motif is a favored target of TB-PFT [12]. Only two of all known C-terminal CaaX -containing proteins have the CVIM CaaX -motif in *T. brucei brucei* (Fig. S1). One CVIM-containing gene, Gene DB# Tb10.70.0590, is a predicted ras-like small GTPase and has been previously characterized in *T. cruzi* [19]. The second, Gene DB# Tb927.10.12440, was annotated as a kinesin-like protein with a predicted molecular weight of 91 kilodaltons. As we had observed a decrease in prenylation of proteins at approximately this molecular weight in protein farnesyl transferase inhibitor (PFTi) -treated *T. brucei* [11] and because no other proteins with a CaaX motif besides Tb927.10.12440 were between 77 and 98 kilodaltons (Fig. S1), we set out to further investigate this protein. Bioinformatics studies reveal this protein contains motifs common to the kinesin family of motor proteins. Due to the presence of the CaaX motif we have named the protein Kinesin^CaaX^. In our present study we demonstrate this protein is farnesylated in *T. brucei* cells and this farnesylation has functional effects. With Kinesin^CaaX^ protein depletion using RNAi we demonstrate the importance of Kinesin^CaaX^ for *T. brucei* proliferation. We characterize tetracycline-induced exogenous tagged Kinesin^CaaX^ expression in *T. brucei* and localize it in the cell and show this localization to be dependent on prenylation. Through our investigation of cell cycle, nucleus and kinetoplast quantitation and immunofluorescence assays we hypothesize an important role for Kinesin^CaaX^ in nuclear and kinetoplast segregation.

## Results

### Kinesin^CaaX^ has motifs consistent with kinesin motor proteins

**Figure 1:**
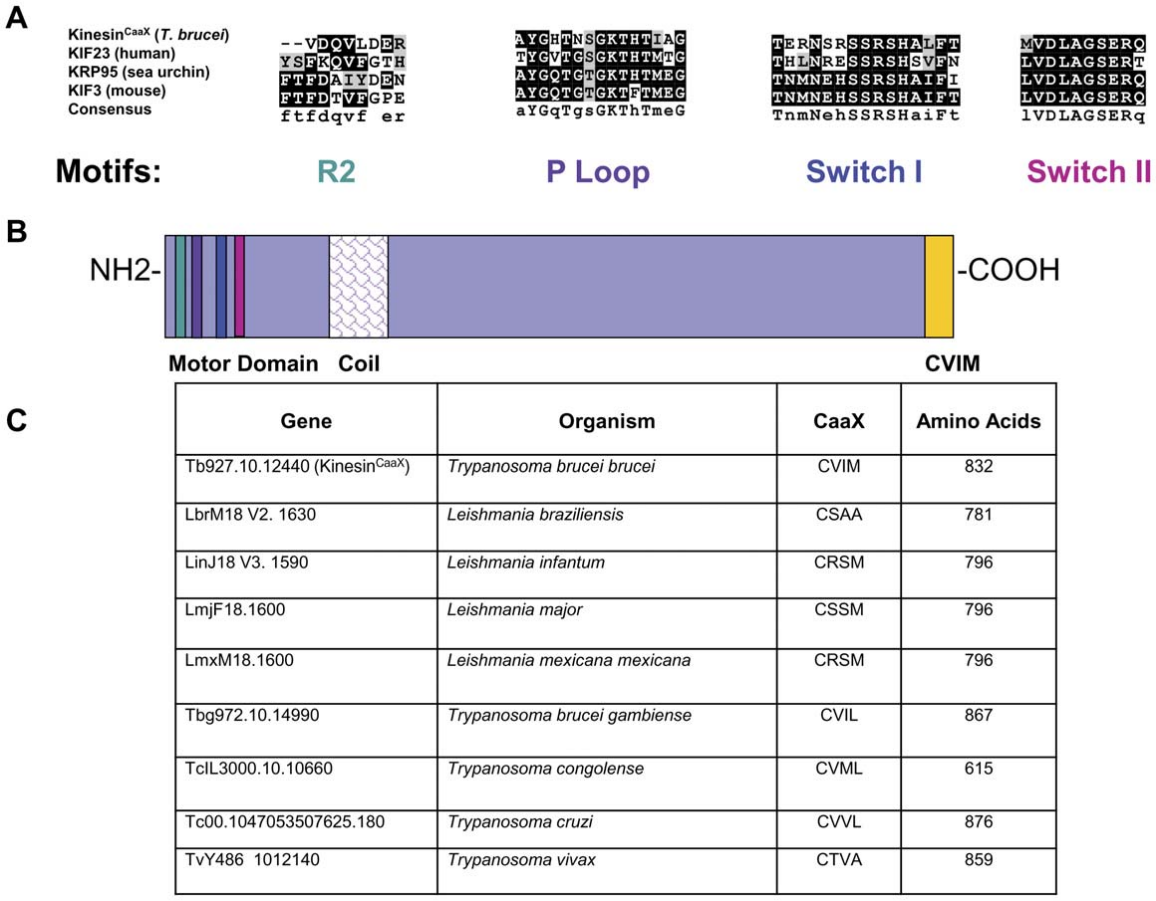
Kinesin^CaaX^ has motifs consistent with plus-end directed kinesin proteins and is conserved in pathogenic kinetoplastids. (**A**) Key kinesin domains are conserved in Tb10.389.1270 when sequences are compared to kinesins in sea urchins, mice and humans. The corresponding GenBank accession numbers for each corresponding protein are *Homo sapiens* NP_612565.1, *Mus musculus* NP_03246, and *Strongylocentrotus purpuratus* P46871. (**B**) Structural features and domain architecture of Kinesin^CaaX^. Kinesin^CaaX^ has 832 residues. Represented are the R2 domain (teal), the phosphate binding loop or P Loop (purple), Switch I (blue) and Switch II (magenta) regions, the coil region (fish scales) that facilitates dimerization, and CaaX motif (yellow) that is predicted to act as a farnesylation signal. (**C**) Comparison of Kinesin^CaaX^ orthologs in other kinetoplastids shows CaaX motif and size conservation in other pathogenic kinetoplastid species.

All motifs common to the kinesin family of motor proteins are present in Kinesin^CaaX^. Kinesins are proteins that convert the chemical energy from ATP into a mechanical force to move along microtubules [22]. Kinesins include a diverse range of molecules organized into nearly 20 families based on the location of the motor domain in the protein and regions of homology outside of the motor domain [23]. Kinesins from various families have diverse functions and may function as monomers, dimers or tetramers [23]. Kinesins are implicated in moving a wide range of cellular macromolecules, termed “cargo”, including vesicles, organelles and chromosomes [23]. Kinesin^CaaX^ cannot be placed in any known kinesin family due to low homology with previously characterized kinesin families outside the motor domain [24]. Kinesin^CaaX^ is similar in domain layout to the canonical kinesin family 1 members that function as homodimers with two large globular motor domains followed by a coiled-coil neck [25]. The globular motor domain interacts with microtubules and the coiled-coil neck is associated with dimerization. Movement along microtubules has been described as stepwise using 8-nm steps in an asymmetric hand-over-hand mechanism [22]. Structural features involved in force generation in the kinesin superfamily are conserved in Kinesin^CaaX^ when compared to known kinesins in sea urchins, mice and humans (Fig. 1A) as shown by MUSCLE alignment [26]. The R2 domain, P Loop, Switch I and the relay helix Switch II [27] domains are conserved in Kinesin^CaaX^. Kinesin^CaaX^ from *T. brucei brucei* has 832 residues with the motor domain located at the amino terminus of the molecule, followed by a coil region that is predicted to form a coiled coil upon dimerization and the CaaX motif at the carboxy terminus (Fig. 1B). Based on the presence of a coil domain and the motor domain at the amino-terminus of the protein, we predict Kinesin^CaaX^ moves towards the plus-end of microtubules as a dimer.

### Kinesin^CaaX^ is conserved in other pathogenic kinetoplastids but not other eukaryotes

Homology studies utilizing OrthoMCL [28] do not detect orthologs to *T. brucei brucei* Kinesin^CaaX^ in higher eukaryotes, but do predict a single orthologous copy in other pathogenic kinetoplastids including *Leishmania braziliensis*, *Leishmania infantum*, *Leishmania major*, *Leishmania mexicana mexicana*, *Trypanosoma brucei gambiense*, *Trypanosoma brucei rhodesiense*, *Trypanosoma congolense*, *Trypanosoma cruzi* and *Trypanosoma vivax* (Fig. 1C, S2 & S3). The orthologous members vary moderately in molecular weight, yet in each case retain the CaaX motif at the carboxy terminus (Fig. 1C). The evolutionary relationship of Kinesin^CaaX^ was analyzed using CLUSTALW Biology Work Bench, Version 3.2 [29]. This analysis demonstrates *T. brucei gambiense* Kinesin^CaaX^ is most closely related to that of *T. brucei brucei* followed by the other *Trypanosoma species* and then the *Leishmania species* (Fig. S3). As Kinesin^CaaX^ is conserved throughout kinetoplastids but not other higher eukaryotes, we hypothesize this kinesin performs a conserved role in kinetoplastid cell biology.

### Kinesin^CaaX^ has an ATP-dependent motor capable of movement with microtubules

Some predicted kinesins in eukaryotic organisms attach to microtubules but do not demonstrate motor activity. Truncated kinesin genes expressing presumably non-motile kinesins have been suggested to be genetic remnants of once functional proteins [30]. Non-motile kinesins may also facilitate other not yet defined microtubule-protein interactions. To test for motor activity in Kinesin^CaaX^, we used an *in vitro* microtubule motility assay using rhodamine-labeled microtubules and time lapse fluorescence microscopy. Truncated recombinant Kinesin^CaaX^ containing the motor domain and the coil dimerization domain (Fig. S4A) led to the processive movement of fluorescent microtubules, in a linear direction, in the presence of ATP (Fig. S4B). Control extracts expressing β-galactosidase, non-motor recombinant protein plus ATP did not lead to the movement of fluorescent microtubules (Fig. S4C). Kinesin^CaaX^ without ATP also did not lead to the movement of fluorescent microtubules (data not shown). Thus, both ATP and the Kinesin^CaaX^ motor domain were required to move microtubules. These studies demonstrate that Kinesin^CaaX^ has motor activity with microtubules in an ATP dependent manner. We hypothesize Kinesin^CaaX^ uses this ATP-dependent motor activity to move cargo along the microtubule network within *T. brucei* cells. As a more sophisticated biophysical study would require a full length protein isolated from *T. brucei*, we proceeded to studies to elucidate the functional significance of Kinesin^CaaX^ in the parasite.

### Reduction of Kinesin^CaaX^ expression in the bloodstream form inhibits growth and contributes to mitotic delay

**Figure 2:**
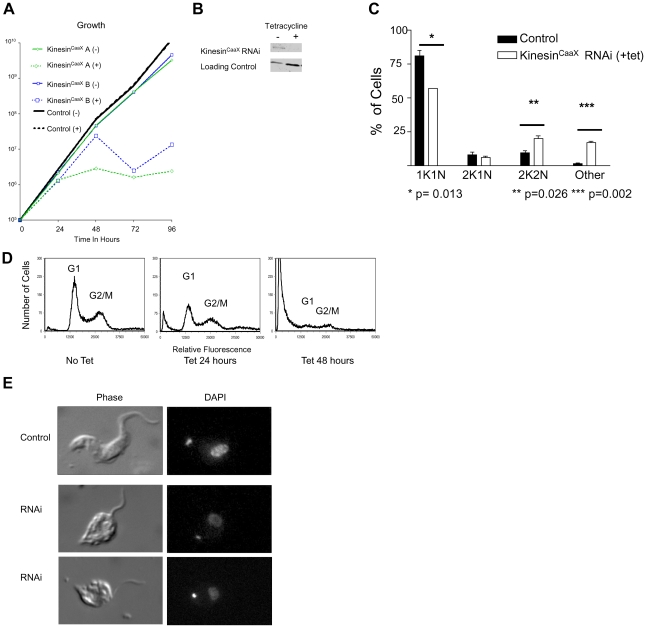
RNAi of Kinesin^CaaX^ in the bloodstream form inhibits growth and contributes to mitotic delay. (**A**) Growth of tetracycline-inducible RNAi Kinesin^CaaX^ knockdown cells with and without tetracycline induction. RNAi cell lines were constructed with the tetracycline-inducible vector to induce double stranded RNA specific to Kinesin^CaaX^. Kinesin^CaaX^ A and B represent two different clones. Untransfected and uninduced (−tet) cells are used as controls. Broken lines indicate tetracycline added to cultures during growth (+). Kinesin^CaaX^ RNAi-induction results in a significant growth defect in both A & B clones. (**B**) Western blot analysis of Clone A whole cell lysates of bloodstream (BSF) probed with rabbit serum from animals immunized with a fragment of recombinant Kinesin^CaaX^. Non-induced (−) and tetracycline induced (+) cells reveal Kinesin^CaaX^ protein levels (91 kDa) were diminished by 48 hours of tetracycline-induction. A 21 kDa non-specific band from the Western Blot serves as a loading control. The same loading ratio of − to + tetracycline protein was confirmed in non-transferred Coomassie stained PAGE lanes (data not shown). (**C**) Fluorescence microscopy of DAPI stained cells demonstrates a shift in kinetoplast and nuclei content of Kinesin^CaaX^-depleted cells. Shown are the mean and SD of 2 experiments at 24 hours after tetracycline induction performed with clone A cells. Control cells are nontransfected controls treated with tetracycline. Similar results were seen with control transfected cells that were not induced with tetracycline (data not shown). Compared with control cells, Kinesin^CaaX^-depleted *T. brucei* cells have a larger proportion of cells with 2 kinetoplasts and 2 nuclei (2K2N) and an increased number of cells with greater than two nuclei (other). Statistical differences were assessed with a Student T Test. (**D**) Flow cytometry reveals Kinesin^CaaX^ depleted *T. brucei* cells, compared with non-induced cells, is dominated by the appearance of an increased number of cells with lower than G1 DNA content, particularly evident at 48 hours after induction. The Y axis represents the number of cells and the X axis is DAPI fluorescence, representing DNA content. The lines represent histograms of cells. Peaks representing G1 and G2/M are indicated. (**E**) Photomicrographs of phase and DAPI-stained *T. brucei* blood-stage cells. Depletion of Kinesin^CaaX^ (RNAi) leads to cell morphology changes with cells demonstrating a rounded cell body and detached flagella compared to the non-induced cells (Control). The RNAi examples represent two typical cells, with altered morphology, observed at 48 hrs after tetracycline induction.

*T. brucei* and other kinetoplastids have a complex life cycle with various developmental forms. Two forms of *T. brucei brucei* can be cultured and studied in the lab; the mammalian bloodstream (trypomastigote) form and the insect (procyclic) form. We utilized RNA interference (RNAi) in order to test the effect of reduced Kinesin^CaaX^ expression on the phenotype of bloodstream trypomastigotes, as trypomastigotes are the forms most relevant to human and animal disease. We cloned a unique portion of the Kinesin^CaaX^ ORF into a tetracycline-inducible construct designed with opposing T7 promoters that induce dsRNA and made stably transfected *T. brucei brucei* bloodstream parasites. Within 72 hours of tetracycline induction of Kinesin^CaaX^ RNAi, a dramatic growth defect occurred in two independently derived clones (Fig. 2A). RT-PCR for Kinesin^CaaX^ mRNA reveals levels from induced with tetracycline were reduced to 18% (Clone A) and 32% (Clone B) of uninduced controls by 48 hours. Levels of Kinesin^CaaX^ mRNA of no-tetracycline controls were similar to quantities observed in non-transfected controls. Further work was carried out with clone A because of its superior RNA reduction after tetracycline induction. Protein levels of Kinesin^CaaX^ clone A were depleted to undetectable levels as shown by Western blot analysis (Fig. 2B). Growth arrest and correlating mRNA and protein depletion of Kinesin^CaaX^ support the hypothesis that Kinesin^CaaX^ is essential for growth in the bloodstream form.

*T. brucei* cells cannot be easily synchronized to study the cell cycle, but kinetoplast and nuclear DNA quantitative analysis by fluorescence microscopy of DAPI stained fixed cells can be used to detect alterations in cell cycle progression. G1 cells are diploid and have 1 kinetoplast and 1 nucleus (1K1N). Kinetoplast replication and division occurs before nuclear DNA replication is completed, such that cells undergoing nuclear DNA synthesis (S phase) can have an elongated kinetoplast (still 1K1N) or 2 kinetoplasts and 1 nucleus (2K1N) [30]. Cells with 2 kinetoplasts and 2 nuclei (2K2N) have completed mitotic division and are in G2 or mitosis (G2/M) phase. Cells with greater than 2K2N have not completed cytokinesis. Quantitative analysis reveals cells undergoing Kinesin^CaaX^ RNAi for 24 hours, compared to uninduced cells, demonstrate a decrease of 1K1N cells from 81% to 57% (p = 0.013 Student T test), an increase in 2K2N cells from 9.5% to 20% (p = 0.026 Student T test) and an increase in of cells with greater than 2K2N (other) 1.5% to 17% (p = 0.002 Student T test)(Fig. 2C). No 1K0N cells were observed. Flow cytometry reveals RNAi depletion of Kinesin^CaaX^ results in a slight proportional increase in G2/M cells and a decrease in G1 cells, at 24 hours (Fig. 2D). However, the bulk of the events at 48 hours have low DAPI fluorescence (Fig. 2D, right panel), suggesting that there is an abundance of cell fragments with reduced DNA, likely representing degenerating and dying cells. 1K1N cells undergoing RNAi at 48 hours have a rounded cell body with detached flagella and the DAPI staining appears weaker than non-induced cells (Fig. 2E). Taken together, these data suggest Kinesin^CaaX^ is essential for growth in the bloodstream form.

### Kinesin^CaaX^ is farnesylated in T. brucei

**Figure 3:**
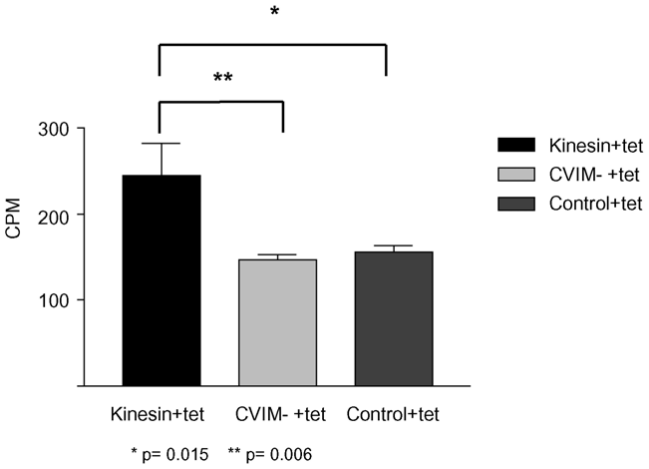
Kinesin^CaaX^ is farnesylated in *T. brucei*. Radioactivity (CPM: counts per minute) found in HA-antibody immunoprecipitates from induced HA-Kinesin^CaaX^ (Kinesin+tet), induced HA-Kinesin^CVIM deletion^ (CVIM- +tet), and control non-transfected cells (Control+tet) extracts from bloodstream form cells. Cells were treated with tetracycline, simvastatin and incubated with ^3^H mevalonolactone for 48 hours. Radioactivity above background is found only in the induced HA-Kinesin^CaaX^ –immunoprecipitates. Whole cell lysates pre-IP were compared using a scintillaton counter to ensure equal lysate concentrations per reaction. Statistical differences were assessed with a Student T Test.

Farnesyl pyrophosphate is used by *T. brucei* protein farnesyl transferase to add farnesyl groups to CaaX-motifs and is generated in a series of enzymatic steps from mevalonate [20]. In the presence of simvastatin, mevalonate synthesis is inhibited, and *T. brucei* cells incorporate scavenged mevalonalactone. Scavenged mevalonalactone is incorporated into farnesyl-pyrophosphate and used to farnesylate proteins [11]. We engineered a tetracycline-inducible plasmid to ectopically express an amino-terminal hemagglutinin-epitope-tagged full length copy of Kinesin^CaaX^ (HA-Kinesin^CaaX^). We made a second construct using site directed mutagenesis, we deleted the twelve nucleotides corresponding to the CVIM (CaaX) from the tetracycline-inducible HA-Kinesin^CaaX^ construct and transfected the plasmid to make a tetracycline inducible HA-Kinesin^CVIM deletion^ expressing *T. brucei* line. Tet-inducible HA-Kinesin^CaaX^, HA-Kinesin^CVIM deletion^ and control (untransfected) *T. brucei* lines were grown in the presence of ^3^H-mevalonalactone, simvastatin and tetracycline. Immunoprecipitation with anti-HA antibodies shows tetracycline-induced HA-Kinesin^CaaX^ immunoprecipitates had a statistically significant increase in radioactive counts per minute (cpm) than control immunoprecipitates (Fig. 3) (p = 0.015, Student T test) and HA-Kinesin^CVIM deletion^ (p = 0.006, Student T test) while Kinesin^CVIM deletion^ did not have a statistically significant difference to the control (p = 0.10, Student T test). These data support our previous finding that TB-PFT adds farnesyl to CVIM peptides and is highly suggestive that CVIM of HA-Kinesin^CaaX^ is farnesylated by TB-PFT in *T. brucei*. This supports the hypothesis that native Kinesin^CaaX^ is farnesylated by TB-PFT in *T. brucei* cells.

### Kinesin^CaaX^ localizes near dividing kinetoplasts and nuclei

**Figure 4:**
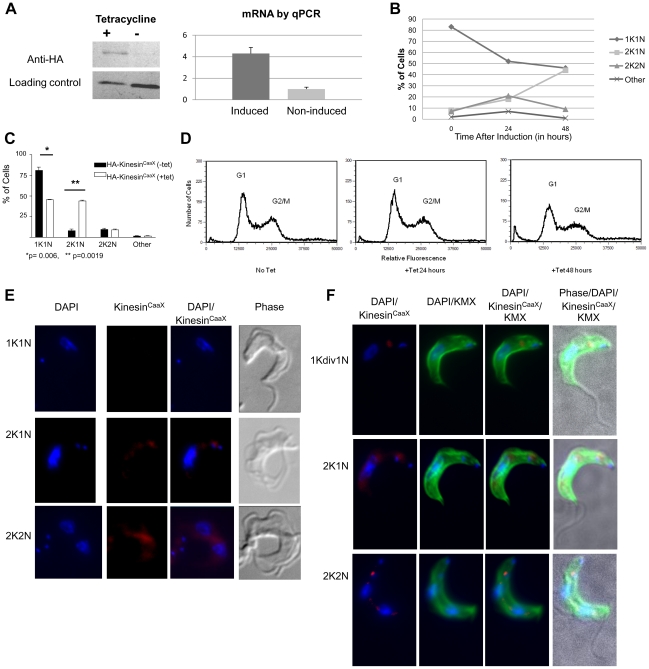
Kinesin^CaaX^ localizes near the nucleus and kinetoplast and is enriched in post-mitotic cells. (**A**) **Left** Western blot of whole cell lysates of bloodstream form (BSF) *T. brucei* tagged HA- Kinesin^CaaX^ cell line either induced (+) or non-induced (−) for 48 hours. While more material is loaded in the (−) non-induced lane, much more HA-Kinesin is observed in the (+) induced lane. **Right** Quantitative PCR (qPCR) showed induced cells had 4.2 times more mRNA for Kinesin^CaaX^ than non-induced controls (error bars are SD). (**B**) DAPI staining and fluorescence microscopy reveals a larger proportion of cells with 2 kinetoplasts and one nucleus (2K1N) and a reduction in 1K1N cells in tetracycline-induced Kinesin^CaaX^ *T. brucei* cells compared with non-induced cells, with the effect becoming prominent at 48 hours (**C**) The mean and SD of 2 experiments at 48 hours. Statistical analysis was performed using a Student T test. (**D**) Ectopic expression of Kinesin^CaaX^ results in a slight decrease in G1 cells and a slight increase in cells between the G1 and G2/M peaks at 48 hours after tetracycline induction. (**E**) Cells were stained with DAPI to stain nuclear and kinetoplast DNA (blue), anti-HA rat mAb primary and polyclonal anti-Rat IgG Cy5.5 (red) secondary 24 hours post-tetracycline induction. Cells with 2K1N and 2K2N DNA content have an increased amount of Kinesin^CaaX^ relative to 1K1N cells. (**F**) Cells were stained with DAPI to stain nuclear and kinetoplast DNA (blue), FITC-anti-mouse IgG to localize the KMX mouse monoclonal antibody (mAb) that binds β- tubulin (green) and anti-HA rat mAb primary and polyclonal anti-rat IgG Cy5.5 (red) secondary that localize HA-Kinesin^CaaX^ 24 hours post tetracycline induction. β-tubulin localization demonstrates these cells have normal morphology. The cell marked 1Kdiv1N has an elongated kinetoplast consistent with a kinetoplast that is undergoing division.

To shed light on the localization of Kinesin^CaaX^, we made an antibody specific to a unique sequence in Kinesin^CaaX^ in *T. brucei* cells and performed immunofluorescence. Despite trying multiple fluorescent signal amplification protocols, expression of native levels of Kinesin^CaaX^ appeared to be too weak to reliably and reproducibly detect by immunofluorescence. We used the HA-Kinesin^CaaX^ exogenous expression lines to study growth and morphology. qPCR shows a 4.3 fold (SD = 0.56) induction of mRNA (Fig. 4A). Western blot using an anti-HA antibody reveals induction of HA-Kinesin^CaaX^ following tetracycline (tet) addition (Fig. 4A). Fluorescence microscopy of DAPI stained cells reveals that induction of HA-Kinesin^CaaX^ results in an increase of cells with the 2K1N configuration from 8% to 44% (p = 0.0019, Student T test) compared to non-induced cells, with a relative decrease in 1K1N cells from 81% to 45.5% by 48 hours (Fig. 4B & 4C) (p = 0.006, Student T test) (Fig. 4C). Flow cytometry analysis on this tetracycline-inducible HA-Kinesin^CaaX^-line reveals a slight decrease in the G1 peak and a slight increase of events between the G1 and G2/M peaks by 48 hours after tetracycline induction (Fig. 4D) consistent with the increase in 2K1N cells. Induced cells have a similar growth rate to control cells and uninduced cells (Fig. S5). Additionally, very few cells with low amounts of DNA (presumably dead cells, Fig. 4D) were seen after Kinesin^CaaX^ induction compared with RNAi induction (Fig. 2D).

As we could not consistently detect native Kinesin^CaaX^ using immunofluorescence, we used the HA-tagged-expression line for localization studies. Exogenously expressed HA-Kinesin^CaaX^ localizes between the nucleus and kinetoplast in a small percentage of 1K1N containing cells but is not present in all 1K1N cells (Fig. 4E & F). In general, 1K1N cells that have detectable HA-Kinesin^CaaX^ have elongated and V-shaped kinetoplasts (Fig. 4F 1Kdiv1N), indicative of initiation of kinetoplast replication [31]. Cells with 2K1N and 2K2N DNA content have more easily detectable HA-Kinesin^CaaX^ relative to 1K1N cells (Fig. 4E & 4F). In 2K1N containing cells, HA-Kinesin^CaaX^ localizes around the nucleus and is enriched near the anterior kinetoplast (Fig. 4E) and in post-mitotic 2K2N cells, Kinesin^CaaX^ is found near the anterior kinetoplast, between the nuclei, and surrounding the nuclei (Fig. 4E). Using the anti-β-tubulin antibody KMX [32] we monitored KMX epitope localization before and after HA-Kinesin^CaaX^ induction (Fig. 4F). KMX localizes to β-tubulin in the microtubule corset of *T. brucei* cells (Fig. 4F). Though the KMX β-tubulin localization looked similar in uninduced cells and induced cells, HA-Kinesin^CaaX^ appeared enriched in the β-tubulin between the nuclei of 2K2N cells (Fig. 4F). These localization data support a role for Kinesin^CaaX^ in separating nuclei and kinetoplasts.

### Kinesin^CaaX^ localization is disrupted with CaaX deletion

**Figure 5:**
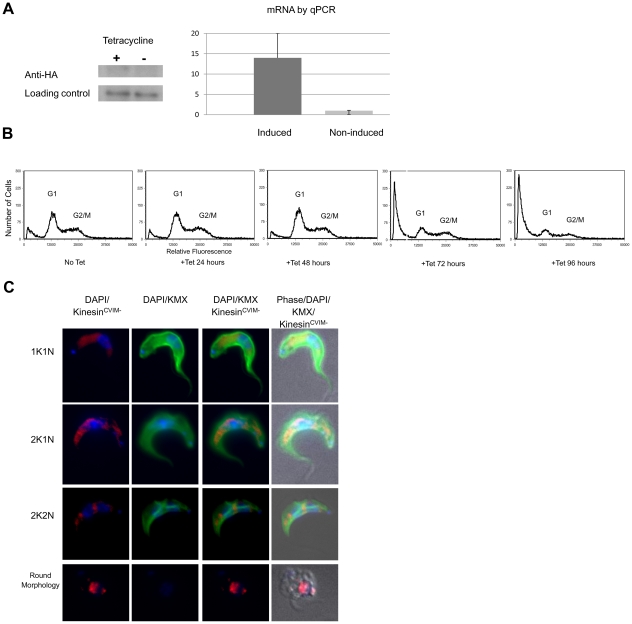
Deletion of CVIM amino acids from Kinesin^CaaX^ results in a more diffuse localization of Kinesin^CVIM deletion^. (**A**) **Left** Western blot of whole cell lysates of bloodstream form (BSF) *T. brucei* tagged HA- Kinesin^CVIM deletion^ cell line either induced (+) or non-induced (−) for 48 hours. **Right** Quantitative PCR (qPCR) showed induced cells had 13.9 fold increase in mRNA for Kinesin^CVIM deletion^ than non-induced controls (error bars are SD). (**B**) Flow cytometry reveals little change at up to 48 hours post tetracycline induction of exogenous HA-Kinesin^CaaX^ expression. Fragmented cells are seen at 72–96, similar to the phenotype observed in RNAi cells at 48 hours. (**C**) Cells tetracycline-induced for 24 hours, fixed and stained with DAPI to stain nuclear and kinetoplast DNA (blue), FITC-anti-mouse IgG to stain mouse anti-β tubulin KMX antibody(green) and anti-HA rat mAb primary and polyclonal anti-IgG Cy5.5 (red) secondary.

Upon tetracycline induction Kinesin^CVIM deletion^ mRNA is induced 13.9 fold (SD = 6.5) as shown by RT-PCR and protein is detected and Western blot analysis (Fig. 5A). HA-Kinesin^CVIM deletion^ expressing-cells demonstrate a similar growth rate as control cells for the first 72 hours and then demonstrate a slowed growth rate by 96 hours post-induction (Fig. S5). Upon tetracycline induction, HA-Kinesin^CVIM deletion^ cells have a very similar nuclear and kinetoplast quantitative profile to control unstimulated cells at 24 and 48 hours (data not shown) consistent with flow cytometry profiles of G1 and G2/M (Fig. 5B). While profiles of the 24 and 48 hour time points after induction are very similar to the non-induced control by 72 hours and at 96 hours after induction, a large population of cells containing less than a G1 complement of DNA becomes apparent, suggestive of dying and degenerating cells. This is consistent with the observed decrease in growth by 96 hours (Fig. S5). Immunofluorescence microscopy shows the HA-Kinesin^CVIM deletion^ has a more diffuse localization in 1K1N, 2K1N and 2K2N cells than the HA-tagged Kinesin^CaaX^ cells at 24 hours post-induction (Fig. 5C). Additionally we observed cells with a rounded cell body some of which disrupted the KMX antibody β-tubulin binding (Fig. 5C). This cell morphology is similar to what is observed in cells undergoing Kinesin^CaaX^ RNAi (Fig. 2E). Thus the exogenous expression of Kinesin^CVIMdeletion^ led to a death phenotype similar to that seen in RNAi depleted cells, but at later time points than that seen with RNAi. Furthermore, Kinesin^CVIM deletion^ was found much more diffusely localized throughout the cells than Kinesin^CaaX^, suggesting the farnesyl group contributes to proper localization.

### Depletion of Kinesin^CaaX^ leads to enhanced sensitivity to protein farnesyl transferase inhibitors but not other classes of drugs

**Figure 6:**
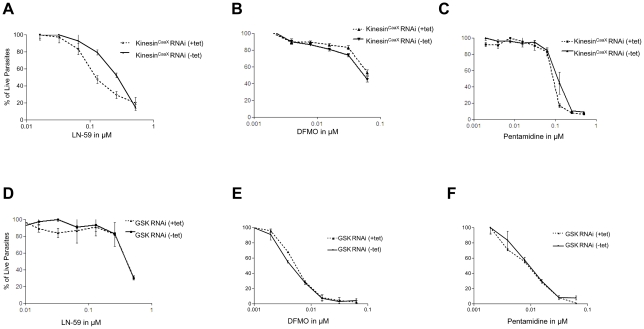
RNAi of Kinesin^CaaX^ show enhanced sensitivity to protein farnesyl transferase inhibitors but not other drugs. (**A**) LN-59 (**B**) DFMO and (**C**) Pentamidine mean % growth (normalized to no drug) in induced and noninduced Kinesin^CaaX^ RNAi cells. (**D**) LN-59 (**E**) DFMO and (**F**) Pentamidine mean % growth in induced and noninduced GSK RNAi line. Values normalized to no drug with error bars representing SD.

Our group has characterized and optimized protein farneysltransferase inhibitor molecules based on the tetrahydroquinoline (THQ) scaffold [33]. LN-59 is a THQ compound (Structure 4 g in reference 32) that has potent activity against mammalian PFT (IC_50_ of 3.2 nM) and *Plasmodium falciparum* PFT (IC_50_ of 1.1 nM) [33]. Additionally LN-59 has activity against TB-PFT (IC_50_ of 35 nM) and against *T. brucei* 427 bloodstream-form parasites in whole cell screens (EC_50_ of 63 nM) suggesting TB-PFT is the target of growth inhibition. In proliferation assays, *T. brucei* depleted of Kinesin^CaaX^ by RNAi had enhanced sensitivity to LN-59 (Fig. 6A). In five independent experiments, effective concentrations of LN-59 to reduce proliferation by 50% (EC50s) were 4.2 fold less in the Kinesin^CaaX^ -depleted cells compared with non-induced controls (SD of 1.0, Student T Test p<0.001). *T. brucei* depleted of Kinesin^CaaX^ by RNAi were not hypersensitive to DFMO (Fig. 6B) or pentamidine (Fig. 6C), compounds that do not act through inhibition of TB-PFT. DFMO and pentamidine were tested three times each, and the same EC50s were observed whether tet-induced to reduce Kinesin^CaaX^ or not. Additionally we tested another RNAi line, which gave similar growth reduction to that of Kinesin^CaaX^ upon tetracycline induction, a glycogen synthase kinase (GSK) RNAi line [34]. *T. brucei* depleted of GSK by RNAi were not hypersensitive to LN-59 (Fig. 6D), DFMO (Fig. 6E), or pentamidine (Fig. 6F). These assays demonstrate that cells with reduced Kinesin^CaaX^ are more sensitive to a PFT inhibitor but not two drugs from other classes. These data demonstrate that depletion of a CaaX-containing molecular target sensitizes *T. brucei* to protein farnesyltransferase inhibition of cell growth.

## Discussion

Our analysis reveals an important role for Kinesin^CaaX^ in the cell growth of *T. brucei*. In this study we have demonstrated Kinesin^CaaX^ has motor activity along microtubules and is essential for growth in the bloodstream form. Kinesin^CaaX^ depleted cells (RNAi-induced cells) demonstrate an increase of cells with less than a G1 complement of DNA, which are presumably dying. Kinesin^CaaX^-depleted cells have more rounded cell bodies compared to control uninduced *T. brucei* cells. This change in morphology could be a direct effect of reduced Kinesin^CaaX^ or an indirect effect of cell death. Supporting that rounded cells were dying, the rounded cells that predominated during RNAi appeared to have weaker DAPI staining, implying their nuclei were undergoing degradation. Exogenous expression cell lines show HA-Kinesin^CaaX^ is highly enriched near the anterior kinetoplast and near the nucleus in 2K1N containing cells and between post-mitotic nuclei in 2K2N containing cells prior to cytokinesis. In our exogenous expression lines, cells with 2K1N and 2K2N content had more easily detectable Kinesin^CaaX^ relative to 1K1N cells perhaps suggesting a role in separation of nuclei and kinetoplasts. HA-Kinesin^CaaX^ was found to overlay β-tubulin in this area between 2 nuclei in 2K2N cells and this localization may also support a role for Kinesin^CaaX^ in the separation of nuclei. It is possible that post-transcriptional regulation of Kinesin^CaaX^ levels, such as protein instability, could be responsible for the reduced detection of Kinesin^CaaX^ in 1K1N cells. Kinesin Family 5 and Family 7 members responsible for chromosome separation in higher organisms are lacking in *T. brucei* [24]. Due to localization and increased abundance of HA-Kinesin^CaaX^ in cells near kinetoplast and nuclear DNA, we hypothesize that Kinesin^CaaX^ may facilitate nuclear and kinetoplast segregation.

Our radiolabeling studies show that HA-Kinesin^CaaX^ but not HA-Kinesin^CVIM deletion^ is a target for prenylation in *T. brucei* cells. To uncover the role of farnesylation in Kinesin^CaaX^, we localized ectopically-expressed HA-Kinesin^CVIM deletion^. HA-Kinesin^CVIM deletion^ expression results in a more diffuse localization pattern compared with HA-Kinesin^CaaX^. This supports the hypothesis that farnesylation helps Kinesin^CaaX^ appropriately localize in the cell. Prolonged ectopic expression of HA-Kinesin^CVIM deletion^ leads to cells with rounded cell bodies and reduced DNA content, a phenotype we also observed in the RNAi experiments. We speculate this apparent increase in cell death occurs due to an accumulation of Kinesin^CVIM deletion^ molecules without prenylation, outcompeting the endogenous Kinesin^CaaX^ for functional interactions. We speculate the delayed 96 hour Kinesin^CVIM deletion^ effect, compared to the early phenotype that is evident at 48 hours in the RNAi experiments, may be due to heterodimers of native Kinesin^CaaX^ and HA-Kinesin^CVIM deletion^ being functional at 48 hours after tet-induction and homodimers of HA-Kinesin^CVIM deletion^ that accumulate 96 hours after tet-induction may not localize or function properly. We hypothesize Kinesin^CaaX^ interacts with cellular cargo via the farnesyl group at the C-terminus, and the motor domain at the N-terminus allows the Kinesin^CaaX^ to move along microtubules of the cell. Future experiments are needed define the cargo of Kinesin^CaaX^.

We also show Kinesin^CaaX^ RNAi cells have an increased sensitivity to a protein farnesyl transferase inhibitor (PFTi) but not other classes of drugs when compared to cells with wildtype levels of Kinesin^CaaX^. Collectively these data suggest that farnesylation of Kinesin^CaaX^ is integral to the function of Kinesin^CaaX^. This is the first study of a molecular target downstream of *T. brucei* protein farnesyl transferase (TB-PFT). While there are many proteins with CaaX motifs in *T. brucei* that may be modified by TB-PFT (Fig. S1), our data, of increased sensitivity to PFTi when Kinesin^CaaX^ is reduced, suggest that inhibition of farnesylation of Kinesin^CaaX^ contributes substantially to the growth inhibition of PFTi-treated *T. brucei*.

An analysis comparing the kinesins of various organisms, including *T. brucei*, was unable to group Kinesin^CaaX^ with other known kinesin family members [35]. In our work, we show the essential motor motifs are intact in Kinesin^CaaX^ (Fig. 1) and demonstrate ATP-dependent motor activity characteristic of kinesins (Fig. S4B). Based on the localization pattern during various stages of the cell cycle, we speculate Kinesin^CaaX^ may be facilitating factors key to kinetoplast and nuclear DNA segregation. Centromeric protein E (CENP-E) is a kinesin in higher eukaryotic organisms that contains a CaaX box, is farnesylated and has been shown to be an N-terminal processive motor protein [36]–[38], all properties similar to Kinesin^CaaX^. CENP-E is a Kinesin-7 family member and is involved in chromosome movement during mitosis and links centromeres to spindle microtubules. Similar to the observed levels in expression of HA-Kinesin^CaaX^, CENP-E has the maximum level during late G2 and minimal levels in G1 [37]. CENP-E localization to kinetochores occurs from early premetaphase through anaphase. CENP-E is important during all phases of mitotic chromosome movement and affects kinetochore-microtubule capture. CENP-E loss of function by interfering RNA results in cell cycle arrest [37]. Additionally the use of protein farnesyl transferase inhibitors has been shown to block the association of CENP-E with microtubules [38]. Currently no CENP-E homolog or any Kinesin-7 family member has been uncovered in the Tritryp genomes by bioinformatic studies [24]. Several other kinesins have recently been characterized in *T. brucei* including the C-terminal Kinesin-13 proteins [39], [40] TbKif13-1 is associated with the nucleus and nuclei in a non cell-cycle dependent manner [40]. Depletion of TbKif13-1 in *T. brucei* results in an increase in G2/M cells by 48 hours and causes altered minichromosome segregation [39].

We speculate Kinesin^CaaX^ may play a role in nuclear and kinetoplast segregation. Kinetochores, the multimeric protein structures that attach at the centromere spindle microtubules to chromosomes during cell division, have not been observed in *T. brucei*. Peripheral microtubules structures that are suggestive of pole-kinetochore microtubules have been observed in *T. brucei*. These peripheral microtubules terminate in electron dense structures and these structures may be serving as conventional kinetochores. However, there is a discrepancy between the number of large chromosomes, 22 in the diploid set of *T. brucei* and the 10 electron dense structures seen in *T. brucei* cells [41]. Minichromosomes are hypothesized to segregate from the microtubule spindle through tracking along microtubules that extend to the poles rather than via a conventional kinetochore attachment [41]. In mammalian cells, the inner kinetochore attaches to the DNA at the centromeric region while the outer kinetochore proteins interact with the spindle microtubules. Bioinformatic analysis of the Tritryp (*T. brucei, L. major, T. cruzi*) genomes does not reveal homologs for most of the proteins of the outer kinetochore including CENP-E, CENP-F, HEC1/Ndc80, Nuf2 or for inner kinetochore components including CENP-C, CENP-G, Cep3p, Mis12, Nde10p and Ctf13p [24]. One outer kinetochore protein, TOG/MOR1 and two inner kinetochore proteins, MCAK and Skp1p have been annotated in the TriTryps [24] yet their roles in *T. brucei* have not been characterized. As Tritryps do not encode homologs to the majority of conventional kinetochore components other proteins must facilitate successful segregation of their DNA. Our work demonstrates the same pattern of accumulation of Kinesin^CaaX^ throughout the cell cycle as seen with other reported studies on CENP-E. Kinesin^CaaX^ and the associated farnesyl group may function in a similar manner as CENP-E creating additional interactions with microtubules at the C-terminus of Kinesin^CaaX^ via the farnesyl group, and thus move microtubules associated with chromosomes, nuclei or kinetoplasts along other microtubules. Alternatively, the farnesyl group may provide a hydrophobic attachment to a membrane target, such as nuclear membranes, allowing nuclear movement during cytokinesis. Thus, CENP-E shares many similarities with Kinesin^CaaX^ and we hypothesize that this kinesin performs functions in trypanosomatids that CENP-E performs in higher eukaryotes.

In preparation of this manuscript, Kinesin^CaaX^ was identified among a list of proteins in a palmitoylation screen [42]. We speculate based on the cysteine conserved three amino acids before the CaaX motif that this protein is both palmitoylated and farnesylated. We note that the cysteine residue three residues upstream of the CaaX motif is also conserved in all pathogenic kinetoplastids (Fig. S2). While we did not remove the cysteine where palmitoylation may occur, palmitoylation in this context usually requires farnesylation which does not occur if the CaaX motif is removed. Thus we hypothesize that our HA-Kinesin^CVIMdeleted^ construct lacks both post-translational modifications and both modifications contribute to proper localization.

In conclusion our work characterizes a kinetoplastid-specific kinesin that has a site for farnesylation, undergoes farneyslation in *T. brucei* and requires farnesylation for proper function. We hypothesize this farnesylation could facilitate interactions with microtubules, as is the case with CENP-E and H-ras and N-ras in mammalian cells, or with other cargo such as the nuclear membrane. Our work suggests inhibition of farnesylation of Kinesin^CaaX^ contributes to the molecular mechanism of growth arrest in PFTi-treated *T. brucei*. This is the first study to demonstrate motor activity of a *T. brucei* kinesin and the first study to characterize farnesylation of a kinesin in *T. brucei*. Further understanding of Kinesin^CaaX^ and of interacting partners of Kinesin^CaaX^ in *T. brucei* and other pathogenic kinetoplasts may be helpful to finding new therapeutic interventions for these parasitic pathogens.

## Materials and Methods

### Cell maintenance

The single marker bloodstream form (BSF) strain *T. brucei brucei* 427 cell line that expresses T7 RNA polymerase and Tet-repressor [43] was maintained in HMI-9 medium containing 10% tetracycline-free fetal bovine serum (Atlanta Biologicals). Cultures were maintained at 37°C with 5% CO_2_ as previously described [39]. Cells were diluted into fresh medium once cell density achieved 2×10^6^ cells/mL.

### Generation of Kinesin^CaaX^ RNAi cell lines

Using the primer prediction algorithm, RNAit [44], primers were selected to minimize off-target effects. The 600 nucleotide sequence was amplified from the full length Kinesin^CaaX^ ORF of the expression plasmid and confirmed to contain Kinesin^CaaX^ by dye deoxy sequencing. The 600 base pair PCR product was analyzed by gel electrophoresis and compared with the predicted product size. The RNAi vector p2T7^TAblue^ [45] (gift from David Horn) was cut to remove the stuffer region and gel purified. The PCR product was then ligated into p2T7^TAblue^ and transformed into *E. coli* DH5alpha. Plasmids were screened by restriction enzyme digestion and verified by dye deoxy sequencing prior to trypanosome transformation. Midlog *T. brucei* (2.5×10^7^) were resuspended in cytomix (120 mM KCl, 0.15 mM CaCl_2_, 10 mM K_2_HPO_4_/KH2PO_4_ pH 7.6, 25 mM HEPES, 2 mM Na_2_EDTA, 5 mM MgCl_2_) containing 12 µg of *Not*I linearized p2T7^TAblue^ DNA. The mixture was electroporated in a 4 mm gap cuvette with 1.6 kV and 24Ω resistance. Cells were resuspended in HMI-9 media. After 6 hours of recovery, selective drug (2.5 µg/mL hygromycin and 2.5 µg/mL G418) was applied and serial dilutions were made into 24-well plates. Transfected cells grew out from limit diluted cells in 6 to 8 days after electroporation.

### Construction of Kinesin^CaaX^ expression strains

The complete open reading frame of the *T. brucei* Kinesin^CaaX^ was amplified using *T. brucei brucei* genomic DNA, strain 427, using primers with an added *Hind*III and *Bam*HI sequences for subcloning and a N terminal double HA tag using primers 5′CCAAAAAGTAAAATTCACTATCCATATGACGTCCCAGACTCTGCCTATCCATATGACGTCCCAGACTCTGCCAAGCTTATGTCGGGTATATATGCG3′ and antisense 5′CCGGCTACATTATTATTACACAGTAATGGCAACCC3′. The HA tag was engineered with the *Aat*I restriction site (GACGTC) to test for insertion. The PCR product was analyzed by gel electrophoresis and gel purified. The expression vector pHD^538^ [46] (gift from Christine Clayton) was cut to remove the stuffer region and gel purified. The PCR product was then ligated into the vector and transformed into *E. coli* DH5alpha. Plasmids were isolated using QIAGEN kits, screened by restriction enzyme digestion, verified by dye deoxy sequencing, analyzed using Vector NTI Suite 9.0 (Invitrogen) and linearized with *Not* I prior to trypanosome transformation. Trypanosome transformation and drug selection were performed as described above.

### Removal of CVIM from Kinesin^CaaX^

Using the pHD^538^ Kinesin^CaaX^ as template we performed site directed mutagenesis using the primers 5′TAAACAGGGTTGCCATTACTAGGGATCCAATTTTCCCC′3

5′ GGGGAAAATTGGATCCCTAGTAATGGCAACCCTGTTTA′3 and the *Pfu* Ultra polymerase to remove the entire CaaX motif, in order to explore the function of Kinesin^CaaX^ when it was not farnesylated as previously described [47]. As the protease that acts after farnesylation removes the VIM residues, we felt that removing the entire CVIM terminus would lead to a better physiologic demonstration of what functionally happens to Kinesin^CaaX^ without prenylation. Amplification conditions were 5 minutes at 95°C, followed by 12 cycles of 1 minute at 95°C, 1 minute at 55°C and 18 minutes at 68°C. The PCR reaction was then *Dpn* I treated overnight to digest the parental plasmid DNA and transformed into *E. coli* XL10-Gold cells (Invitrogen). Plasmids were screened by restriction enzyme digestion, verified by dye deoxy sequencing to have the desired deletion, analyzed using Vector NTI Suite 9.0 (Invitrogen) and linearized with *Not* I prior to trypanosome transformation as described above for Kinesin^CaaX^.

### RT-PCR

Total RNA was extracted from mid-logarithmic phase cultures of *T. brucei* using the Qiagen RNeasy Mini kit. mRNA was isolated using the Qiagen Oligotex system from 10 µg of total RNA. Reverse transcription using 25 ng of mRNA per reaction using random hexamers with TaqMan Reverse Transcription Reagents (Applied Biosystems) yielded cDNA. Samples without reverse transcriptase were included to confirm the absence of contaminating genomic DNA. cDNA reactions were diluted two-fold and amplified in 25 µL reactions containing 2.5 µL of each cDNA, 5 µL each of 1.5 µM sense and antisense primers, and 12.5 µL SYBR Green PCR Master Mix (Applied Biosystems) in 96-well plates using the Opticon 2 Real-Time PCR Detection System (BioRad Life Sciences). Amplification conditions were 2 minutes at 50°C and 10 minutes at 95°C, followed by 45 cycles of 15 seconds at 95°C, 1 minute at 55°C and 1 minute 30 seconds at 60°C, 1 second at 80°C. Using the primers corresponding to the 254–353 basepair region, sense 5′-GCTCCAACCGAACGTAACTC-3′ and antisense 5′-CACTTCCCGCTAAGTCAACC-3′. Additionally tubulin primers used as a control were sense 5′-TTCCGCACCCTGAAACTGA-3′ and antisense 5′-TGACGCCGGACACAACAG-3′. Relative changes were determined after normalization to β-tubulin mRNA and expressed as relative mRNA abundance from the respective control cells.

### Production of Polyclonal antibody against Kinesin^CaaX^ protein

The *T. brucei* Kinesin^CaaX^ amino acids 409–525 corresponding to the least similar region to other *T. brucei* proteins, as analyzed by BLAST [48], was amplified using the full length gene from the expression plasmids as template using the LIC primers sense 5′CTCACCACCACCACCACCAT3′ and antisense 5′ATCCTATCTTACTCACTTA3′ coding for an N-terminally 6Xhistidine-tagged Kinesin^CaaX^ fragment. Nine amino acid segments were checked against the *T. brucei* genome using BLAST to ensure the specificity to Kinesin^CaaX^. The PCR product was then put into the LIC expression vector Bg1861 and transformed into *E. coli* NovaBlue cells (Novagen). Plasmids were screened by restriction enzyme digestion, verified by dye deoxy sequencing and analyzed using Vector NTI Suite 9.0 (Invitrogen). The recombinant plasmid was then transformed into *E. coli* BL21cells (Promega) and grown and induced using IPTG for protein production. Bacteria were harvested by centrifugation at 2,500 g for 10 minutes at 4°C. Bacterial cells were lysed in lysis buffer (50 mM Tris, 100 mM NaCl, 1 mM MgCl_2_, and Protease Inhibitor Cocktail (Sigma-P8430), pH 7.0) and purified using Nickel-NTA resin (QIAGEN). The purified recombinant protein was dialyzed against PBS. The purified recombinant protein was sequenced using trypsin digestion and mass spectrometry to verify peptide purity. The purified recombinant protein was used to raise rabbit polyclonal antibodies (R&R Rabbitry). Rabbit serum was preblocked with *E. coli* lysate to reduce nonspecific binding prior to Western blot.

### Western Blot

Western blots were performed using 12 µg of total protein induced or noninduced *T. brucei* cultures run on 12% SDS PAGE gels and transferred to PVDF membrane.

Membranes were blocked with 5% nonfat dry milk in Tris buffered saline with 0.1% Tween 20 (TBS-T) on an orbital shaker overnight at 4°C. Rabbit serum was preabsorbed with *E. coli* lysates overnight. Preabsorbed rabbit serum was diluted 1∶500 in blocking buffer and incubated with the membranes on a shaker for 1 hour at RT. Membranes were washed three times with TBS-T for 10 minutes. Next anti-rabbit secondary antibody conjugated to HRP (Pierce) was added and incubated on a shaker for 1 hour at RT. The membrane was rinsed with TBS-T three times with TBS-T for 10 minutes and developed using ECL Plus (Amersham). Anti-HA Westerns were performed with 3F10 (Roche) 1∶1000 as a primary antibody and secondary anti-Rat antibody, ab6517 (abcam), 1∶15∶000.

### Immunofluorescense microscopy

Cells were washed with PBS, applied to poly-lysine-coated Teflon slides and allowed to adhere. Cells were fixed with 4% paraformaldehyde (PFA) for 5 minutes, washed three times with PBS and permeabilized with 0.05% Triton X at room temperature. Samples were then blocked overnight at 4°C with 5% BSA supplemented with 5% goat serum. The following antibodies were used: The KMX-1 β-tubulin monoclonal antibody [32] (gift from Keith Gull); the High Affinity anti-HA rat mAb 3F10 (Roche); goat polyclonal to rat IgG-H&L Cy5.5 (abcam), FITC-conjugated anti-rabbit IgG (Sigma-Aldrich), anti-mouse IgG 568 (abcam).

Fixed cells were incubated with primary antibodies at room temperature for 60 minutes, washed three times and incubated with FITC-conjugated and Cy5.5 conjugated antibodies for another 60 minutes. After washing three more times, the cells were stained with 1.0 µg/uL of 4, 6-diamino-2-phenlyindole (DAPI) and the slides were mounted with Fluoromount G (Sigma-Aldrich). Cells were examined using a Nikon TE2000 inverted microscope equipped with a Photometrics CoolSNAP HQ2 camera for fluorescence and phase contrast images. Images were analyzed using SoftwoRX software and processed for publication using Adobe Photoshop (Adobe Systems Inc., San Jose, CA) and ImageJ. Secondary antibody only controls were performed and gave only background, low-level fluorescence which was used to set the software for signal detection.

### Flow cytometry

Bloodstream form cells were inoculated at 1×10^5^/mL and allowed to grow. The cultures were harvested at various time points, fixed with 4% paraformaldehyde for five minutes, washed once with PBS and permeabilized with 0.05% Triton X. Lastly DAPI was added and cells were analyzed with an Influx analytical flow cytometer (BD Biosciences). Histograms were created using the FCS Express Version 3 software.

### Drug Assays

*T. brucei* Kinesin^CaaX^ RNAi line cells were inoculated into 96-well plates at 2.5×10^4^/well. To induce dsRNA, 1 µg/mL tetracycline was added and cells were allowed to grow with various concentrations of the LN-59 protein farnesyl transferase inhibitor drug for 48 hours followed by Alamar Blue analysis to follow cell growth as previously described [43]. Pentamidine and difluoromethyl ornithine or DFMO (Sigma-Aldrich) were used as control drugs and compared to cells in media alone. Experiments were done in triplicate. Best fit curves and EC50 values were fitted using Prism 3.0 (Graphpad) and Kaleidagraph 4.0 (Synergy Software). Drug concentrations are expressed in µM.

### Prenylation Determination by Radio-labeling

Double HA-tagged Kinesin^CaaX^ expressing, double HA-tagged Kinesin^CVIM deletion^ and control *T. brucei* cell lines were plated into 6-well plates at 1×10^6^/well and treated with 1 µg/mL tetracycline, 1.25 µM simvastatin and 6.7 µM (100 µCi) [^3^H]-mevalonolactone. Cells were incubated for 48 hours and then processed for immunoprecipitations. [^3^H]-counts were detected using the MicroBeta Scintillation detector (MicroBeta TriLux, Perkin Elmer).

### Immunoprecipitation

*T. brucei* cells were incubated in lysis buffer (25 mM Tris-Cl, pH 7.6, 100 mM NaCl, 1% Nonidet P-40, 1 mM dithiothreitol and protease inhibitor cocktail) for 30 minutes on ice and cleared by centrifugation. The cleared lysate was pre-incubated with protein G Sepharose beads at 4°C for 1 hour. Beads were removed by centrifugation and the bead-cleared supernatant was incubated with anti-HA polyclonal antibody and protein G Sepharose beads at 4°C for two hours. The immune-precipitates collected were then analyzed in the MicroBeta Scintillation detector (MicroBeta TriLux, Perkin Elmer).

### Supplemental Materials and Methods

#### Expression and purification of active Kinesin^CaaX^ in *E. coli*

The *T. brucei* Kinesin^CaaX^ motor domain was amplified using the full length gene from the expression plasmids as template and the ligation independent cloning (LIC) primers 5′CTCACCACCACCACCACCATATGTCAGAGGTACTCGATGCGC 3′ and antisense 5′ATCCTATCTTACTCACTTACTCACTTGCAAGAGTACCACTAAG 3′ coding for a N-terminally 6Xhistidine-tagged Kinesin^CaaX^ motor domain protein. The PCR product was placed into the LIC expression vector Bg1861 [49] and transformed into *E. coli* NovaBlue cells (Novagen). Plasmids were screened by restriction enzyme digestion, checked by dye deoxy sequencing, and the sequence verified using Vector NTI Suite 9.0 (Invitrogen). The recombinant plasmid was then transformed into *E. coli* BL21cells (Promega) and grown and induced using IPTG for protein production. Bacteria were harvested by centrifugation at 2,500 g for 10 minutes at 4°C. Bacterial cells were lysed in lysis buffer (100 mM NaCl, 1 mM MgCl_2_, 1 mM ATP, lysozyme and Protease Inhibitor Cocktail (Sigma-P8430), pH 7.0) and soluble lysates prepared by centrifugation at 14,000× g for 10 minutes. Soluble lysates were used in the motility assay as described below.

#### Gliding Motility Assays

Fluorescent microtubules (MT) were prepared using an 80∶1 ratio of unlabeled bovine-to Alexa 568-labeled bovine tubulin, BRB80 buffer (80 mM Pipes/KOH, pH 6.9, 1 mM EGTA, 1 mM MgCl_2_), 4 mM MgCl_2_, 1 mM GTP, and 5% DMSO at 37°C. After 30 minutes the MT polymers, 5–20 µm in length were stabilized by diluting 200-fold into room-temperature BRB80 containing 10 µM taxol. Microscope slide flow cells were made using a microscope slide fitted with two pieces of double sided tape with 1 cm gap and a coverslip. Motility buffer (BRB80 supplemented with 0.2 mg/mL casein, 20 µM D-glucose, 0.02 mg/mL glucose oxidase, 0.008 mg/mL catalase, 0.5% 2-mercaptoethanol and 32 nM of the final polymerized microtubule reaction) with and without+ 1 mM MgATP was added to the microscope slide coated with *E. coli* soluble lysates containing the recombinant Kinesin^CaaX^ motor protein or the β-galactosidase non-motor control. Time lapse photography was taken every 4 seconds, with a one second exposure, using a Nikon TE2000 inverted microscope, 100× objective, with a Photometrics CoolSNAP HQ2 camera for fluorescence images. Images were analyzed using SoftwoRX software and processed for publication using Adobe Photoshop (Adobe Systems Inc.).

## Supporting Information

Figure S1 **List of CaaX motif containing proteins in** ***T. brucei*** **.** Gene ID, putative name and CaaX motif are listed.
(PDF)

Figure S2 **Kinesin^CaaX^ is conserved in other pathogenic kinetoplastids.** Shown are multiple sequence alignments using MUSCLE version 3.7 [23]. LmxM18.1600 from *Leishmania mexicana mexicana*, LbrM18_V2.1630 from *Leishmania braziliensis*, LinJ18_V3.1590 from *Leishmania infantum*, LmjF18.1600 from *Leishmania major*, Tc00.1047053507625.180 from *Trypanosoma cruzi*, TvY486_1012140 from *Trypanosoma vivax*, TcIL3000.10.10660 from *Trypanosoma congolense*, Tbg972.10.14990 from *Trypanosoma brucei gambiense*, and Tb927.10.12440, herein referred to as Kinesin^CaaX^, from *Trypanosoma brucei brucei*.
(PDF)

Figure S3 **Dendrogram of Kinesin^CaaX^ and orthologs from other pathogenic kinetoplastids.** The evolutionary relationship of Kinesin^CaaX^ was analyzed using Biology Work Bench, Version 3.2, DRAWGRAM and PHYLIP (Phylogeny Inference Package) version 3.5c [26]. This analysis demonstrates the *T. brucei gambiense* homolog to be most closely related to *T. brucei brucei* Kinesin^CaaX^ followed by the other *Trypanosoma species* and then the *Leishmania species*. For gene ID and species key, see Legend of Figure S2. Horizontal distances represent relative genetic distance.
(PDF)

Figure S4 **Kinesin^CaaX^ has motor activity in the presence of ATP** ***in vitro*** **.** (**A**) Recombinant His-tagged truncated N-terminal 340 AA-construct of Kinesin^CaaX^ restricted to the predicted motor domain and the coil region predicted to facilitate dimerization. (**B**) Coomassie stained PAGE showing *E.coli* soluble lysate used for the motor assays in (C) with the increased band at the expected molecular weight (44 kDa) for Kinesin^CaaX^ motor-domain upon IPTG jnduction (+). (**C**) An *in vitro* gliding motility assay using truncated Kinesin^CaaX^ and fluorescent microtubules. Lysates containing recombinant truncated Kinesin^CaaX^ was allowed to adhere to glass microscope slides. Next rhodamine-labeled microtubules were added with or without ATP to the slide and microtubule motility was monitored by time-lapse fluorescence microscopy. Selected frames from time lapse microscopy are represented. The positions of rhodamine-labeled microtubules over time are marked with arrows. (**D**) Control cell lysates containing recombinant β-galactosidase non-motor protein control showed no microtubule movement in the presence of ATP. The positions of two stationary microtubules are marked with arrows.
(TIF)

Figure S5 **Growth rate of** ***T. brucei*** **exogenously expressing Kinesin^CaaX^ is not affected after induction but Kinesin^CVIMdeletion^ induced cells begin to slow their growth rate at 96 hrs.** Shown are growth curves of blood-stream *T. brucei* parasites grown either with (+) or without (−) tetracycline induction. Control parasites are “Single Marker” parasites without an exogenous expression plasmid. Kinesin^CaaX^ and Kinesin^CVIMdeletion^ are parasites transfected with the exogenous expression vector expressing these proteins under control of tetracycline induction. Parasite numbers are shown on the Y axis and time after tetracycline induction in hours is shown on the X axis. The experiment shown represents a single clone from each transfection but the same results were also observed with a second clone from each transfection (data not shown).
(TIF)
