# Supplementary material for: An Essential Farnesylated Kinesin in Trypanosoma brucei
Source: PLoS One. 2011 Nov 2;6(11):e26508. doi: 10.1371/journal.pone.0026508 (PMC3206815; doi:10.1371/journal.pone.0026508)
Supplement: Figure S1 — List of CaaX motif containing proteins in T. brucei . Gene ID, putative name and CaaX motif are listed. (PDF) [file pone.0026508.s001.pdf]

Figure S1:

Gene ID	Putative Name	CaaX	Mass
**Tb927.10.12440 Kinesin^{CaaX}**	**kinesin-like protein**	**CVIM**	**91**
Tb10.70.0590	ras-like small GTPase	CVIM	44
Tb10.70.6010	syntaxin	CVFS	26
Tb10.70.5440	DNAJ	CTQQ	47
Tb10.70.5100	lysosomal alpha-mannosidase precursor	CTGV	112
Tb10.70.4880	eukaryotic translation initiation factor 5	CVAA	43
Tb10.70.4300	U2 splicing auxiliary factor	CPLK	29
Tb10.70.4100	protein kinase	CIIM	99
Tb10.70.2270	protein phosphatase 2C-like	CSER	32
Tb927.10.6070	universal minicircle sequence binding protein (UMSBP)	CPVK	15
Tb10.70.0350	serine-threonine protein phosphatase, 2b catalytic subunit A2	CEGA	44
Tb10.6k15.1990	RAB1 small G-protein	CSVM	25
Tb10.6k15.1790	rab-like GTPase activating protein	CQQL	39
Tb10.389.1800	vesicle-associated membrane protein (VAMP); syntaxin-like protein	CTLM	33
Tb10.61.1330	nucleosome assembly protein	CKHQ	41
Tb11.01.1160	lipoate protein ligase	CELE	30
Tb11.01.1740	2-oxoglutarate dehydrogenase E1 component	CVFS	113

Tb11.01.2530	kinesin-like protein	CVTM	70
Tb11.01.3915	RNA-binding protein	CITA	15
Tb11.01.4450	cyclin-1; serine peptidase family S51, peptidase E	CAPR	42
Tb11.01.6170	cleavage and polyadenylation specificity factor-like protein	CGLF	159
Tb11.01.6250	expression site-associated gene 11 (ESAG 11) protein	CTVL	42
Tb11.47.0002	phosphatidlyinositol (3,5) kinase	CSLA	160
Tb11.02.1380	TRYPARP actin-like protein	CKCQ	48
Tb11.02.3850	TBRLP GTP binding protein	CTML	25
Tb11.02.5060	SNF2/RAD54 related DNA helicase	CPTV	107
Tb11.02.5470	vacuolar type H+ ATPase subunit	CDSS	26
Tb11.02.5700	endonuclease/exonuclease/phosphatase	CLSS	60
Tb11.03.0250	CYPA cyclophilin a	CGQL	19
Tb11.24.0007	variant surface glycoprotein (VSG, atypical)	CSIL	52
Tb927.1.5330	variant surface glycoprotein (VSG)	CKDF	57
Tb927.2.5160/30J2.30	chaperone protein DnaJ	CTQQ	44
Tb927.2.5800/1F7.270	SBPase sedoheptulose-1,7-bisphosphatase	CSKL	36
Tb03.6N20.110	variant surface glycoprotein (VSG)	CFLF	61
Tb927.3.5230	DNA repair protein	CNDE	50
Tb04.2L9.1220	protein kinase	CHCM	76
Tb04.1D20.140	divalent cation tolerance protein	CSCR	13
Tb04.3M17.290	metal-ion transporter	CGTV	51

Tb04.3M17.800	phosphoglycan beta 1,3 galactosyltransferase	CFQH	44
Tb05.30H13.730	mitochondrial carrier protein	CAPA	34
Tb05.45E22.780	protein kinase	CEEG	39
Tb05.25P15.370	variant surface glycoprotein (VSG)	CLIL	58
Tb927.6.5020	CYC7 cyclin 7, putative; CYC2-like cyclin	CLYM	25
Tb07.43M14.520	heat shock protein DnaJ	CTAQ	36
Tb07.26A24.370	cyclophilin-type peptidyl-prolyl cis-trans isomerase	CGVL	19
Tb08.26A17.100	(H+)-ATPase G subunit	CQDN	13
Tb08.28F14.330	mannosyl-oligosaccharide 1,2-alpha-mannosidase IB	CFFF	49
Tb08.11J15.1190	protein tyrosine phosphatase	CAIM	19
Tb08.10K10.800	peptidase, metallo-peptidase, Clan ME, Family M16	CRTF	119
Tb09.160.2210	glutaredoxin-like protein	CRDL	24
Tb09.211.3650	phospholipase A2-like protein	CSTC	50
Tb09.211.3680	chaperone protein DNAJ	CVHQ	49
Tb09.211.4610	vesicle-associated membrane protein	CTVM	23
Tb09.244.2070	GTP-binding protein	CCCQ	31
Tb09.244.0250	variant surface glycoprotein (VSG)	CLLL	53
Tb11.1130	variant surface glycoprotein (VSG)	CKDG	50
