# Supplementary material for: An Essential Farnesylated Kinesin in Trypanosoma brucei
Source: PLoS One. 2011 Nov 2;6(11):e26508. doi: 10.1371/journal.pone.0026508 (PMC3206815; doi:10.1371/journal.pone.0026508)
Supplement: Figure S2 — KinesinCaaX is conserved in other pathogenic kinetoplastids. Shown are multiple sequence alignments using MUSCLE version 3.7 [23]. LmxM18.1600 from Leishmania mexicana mexicana, LbrM18_V2.1630 from Leishmania braziliensis, LinJ18_V3.1590 from Leishmania infantum, LmjF18.1600 from Leishmania major, Tc00.1047053507625.180 from Trypanosoma cruzi, TvY486_1012140 from Trypanosoma vivax, TcIL3000.10.10660 from Trypanosoma congolense, Tbg972.10.14990 from Trypanosoma brucei gambiense, and Tb927.10.12440, herein referred to as KinesinCaaX, from Trypanosoma brucei brucei. (PDF) [file pone.0026508.s002.pdf]

Figure S2:

Multiple sequence alignment using MUSCLE version 3.7.

```
lbra|LbrM18_V2.1630            MSQRRVEVAVRVRPNTELCRNSCVSL-SHATHHISLQDNSGVNALAGRRSYKEDFLFDEH
lmex|LmxM18.1600               MSQRRVEVAVRVRPDTESRHNSCVSL-NHATRRITAQDDIGANVLAGRRSYKEDFLFDEH
linf|LinJ18_V3.1590            MSQRRVEVAVRVRPDTESRHNSCVSL-NHATRHISAQDDTGANALTGRRSYKEDFLFDEH
lmaj|LmjF18.1600               MSQRRVEVAVRVRPDTESRHNSCVSL-NHATRHISTQDDTGANALVGRRFYKEDFLFDEH
tcru|Tc00.1047053507625.180    -MGRRVQLVIRLRPGEGGA--SCVSV-GVNKKEITVVDLSGTHASSTPREFHVDQVVQED
tviv|TvY486_1012140            -MDRRVQVVVRTRPSEGGQ--SCVSI-SSEGKEVVVSDVGAVYGNCVPRSFYVDCSVYAN
tcon|TcIL3000.10.10660         ------------------------------------------------------------
tbrg|Tbg972.10.14990           -MGRRTHVAVRLRPSEGSP--TCVDVKDDKGKEVVVADMSGIYAISIPHIFRVDQVLDER
tbru|Tb927.10.12440            --------------------------------------MSGIYAISIPHIFRVDQVLDER


lbra|LbrM18_V2.1630            VSNRAVFEKLVLQRMLAATPEHPDTLCFLAYGHTSSGKTYTIGGSEKEPGILALCVEELL
lmex|LmxM18.1600               VSNRAVFEELVLQKMRAATPEHPDTLCFLAYGHTSSGKTYTIAGSEQEPGILALCVEELL
linf|LinJ18_V3.1590            VSNRAVFEELVLQKMRAATPEHPDTLCFLAYGHTSSGKTYTIAGSEQEPGILALCVEELL
lmaj|LmjF18.1600               VSNRAVFEELVLQKMRAATPEHPDTLCFLAYGHTSSGKTYTIAGSEQEPGILALCVEELL
tcru|Tc00.1047053507625.180    CGNEELFRSLVLGRIRSSA-DEPDTSCFLAYGHTSSGKTHSIAGTDKEPGLLTLSAEALL
tviv|TvY486_1012140            CTNEKLFEFLVNDRIRTSV-DSPDTSCFLAYGHTNSGKTHTIAGCRREPGLLSLAARKVL
tcon|TcIL3000.10.10660         ------------------------------------------------------------
tbrg|Tbg972.10.14990           CSNEELFEKLVYDRILSSA-EQPDTSCFLAYGHTNSGKTHTIAGGRREAGLLSLAARAVL
tbru|Tb927.10.12440            CSNEELFEKLVYDRILSSA-EQPDTSCFLAYGHTNSGKTHTIAGGRREAGLLSLAARAVL


lbra|LbrM18_V2.1630            RGEGVVEVAMLEVYLESVNDLLAHGEPRHIRRRQGPLGPVIVVEGLTTCSLTSVQQWNAV
lmex|LmxM18.1600               RGEGVVEVAMLEVYLESVNDLLAHGEPRHIRRRQGMRGPVIVVEGLTTCSLTSVEQWNAV
linf|LinJ18_V3.1590            RGEGVVEVAMLEVYLESVNDLLAHGEPRHIRRRQGMQGPVIVVEGLTTCSLTSVEQWNAV
lmaj|LmjF18.1600               RGEGVVEVAMLEVYLESVNDLLAHGEPRHIRRRQGMQGPVIVVEGLTTCSLTSVEQWNAV
tcru|Tc00.1047053507625.180    HTYGVAEVAMLEVYGESVHDLLAQGERRMIRRRAGPDGTVIMVENLTSCSLTSMEEWKAL
tviv|TvY486_1012140            ETHGVLEVSMLEVYGESVHDLLARGEKRFIRRRSSPEGVVIVVENLTCCSLSTLQEWEAV
tcon|TcIL3000.10.10660         ------------------------------------------------------------
tbrg|Tbg972.10.14990           DMYGYLEVAMIEVYGENVHDLLAQGERRLIRRRAGPDGVAIIVEDLTTCSLSTLQEWEAV
tbru|Tb927.10.12440            DMYGYLEVAMIEVYGENVHDLLAQGERRLIRRRAGPDGVAIIVEDLTTCSLSTLQEWEAV


lbra|LbrM18_V2.1630            AAYSMSSRRTAPTERNPRSSRSHAIFTIKSRGVRLCFVDLAGSERQTVFSPQLNKESISI
lmex|LmxM18.1600               AAYGMSSRRTAPTERNSRSSRSHAIFTIKSRGVRLCFVDLAGSERQTVFSPQLNKESISI
linf|LinJ18_V3.1590            AAYGMSSRRTAPTERNPRSSRSHAIFTIKSRGVRLCFVDLAGSERQTVFSPQLNKESISI
lmaj|LmjF18.1600               AAYGMNSRRTAPTERNPRSSRSHAIFTIKSRGVRLCFVDLAGSERQTVFSPQLNKESISI
tcru|Tc00.1047053507625.180    SEFGMLARRTAPTERNSRSSRSHAIFTIKTRGMRLCLVDLAGSERQTTYSPQLNKESIAI
tviv|TvY486_1012140            SAYGMGTRRTAPTDRNSRSSRSHAIFTIKSHGVRLCMVDLAGSERQTTYSPQLNKESIAI
tcon|TcIL3000.10.10660         ------------------------------------------------------------
tbrg|Tbg972.10.14990           SAFGMETRRTAPTERNSRSSRSHALFTIKSRGLRLCMVDLAGSERQTTYSPQLNSESIAI
tbru|Tb927.10.12440            SAFGMETRRTAPTERNSRSSRSHALFTIKSRGLRLCMVDLAGSERQTTYSPQLNSESIAI


lbra|LbrM18_V2.1630            NKSLSRLSTVLEALSNQRVTQDGARPYVNFRDTTLTVLLQRYLTGSSMTTFLACVHPSAD
lmex|LmxM18.1600               NKSLSRLSTVLEALSNQRVAQDGTRSYVNFRDTTLTVLLQRYLTGASMTTFLACVHPSAD
linf|LinJ18_V3.1590            NKSLSRLSTVLEALSNQRVAQDGTRSYVNFRDTTLTVLLQRYLTGASMTTFLACVHPSAD
lmaj|LmjF18.1600               NKSLSRLSTVLEALSNQKVAQDGTRSYVNFRDTTLTVLLQRYLTGASMTTFLACVHPSAD
```

```
tcru|Tc00.1047053507625.180      NKSLSRLSTVLEALSSARMKSDGSTSYVNFRDTTLTVLLQRYLSGASMTVFLACIHPDTA
tviv|TvY486_1012140              NKSLSRLSTVLEALSTTRKRSDGTCSYVNFRDTTLTVLLQRYLCGSSMTVFLACIHPDVQ
tcon|TcIL3000.10.10660           -------STVLEALSTMRRKPDGTSSYVNFRDTTLTVLLQRYLCGASMTVFVACIHPDVQ
tbrg|Tbg972.10.14990             NKSLSRLSTVLEALSTIRRRPDGSSSYVNFRDTTLTVLLQRYLCGASMTVFVACIHPDVQ
tbru|Tb927.10.12440              NKSLSRLSTVLEALSTIRRRPDGSSSYVNFRDTTLTVLLQRYLCGASMTVFVACIHPDVQ
                                        ********. .   **: .***************** *:***.*:**:**..

lbra|LbrM18_V2.1630              YYQETLSTLRYTQRLKRIRTRVTKVDEVE-WSEMKTSEHQVLLDELTRLREQMK------
lmex|LmxM18.1600                 YYQETLSTLRYTQRLKRIRTCITKTDEGE-WSGLKVSEHQVLLDELTRLREQMK------
linf|LinJ18_V3.1590              YYQETLSTLRYTQRLKRIRTRVTKVDEGE-WSGMRVSEHQVLLDELNRLREQMK------
lmaj|LmjF18.1600                 YYQETLSTLRYTQRLKRIRTRVTKADEGE-WSGMRVSEHQVLLDELSRLREQVK------
tcru|Tc00.1047053507625.180      FYYETMSTMRYTQRLKCINTKNAPKKPNEDMSLFQLGENQKLLEELMILRRIVGRQHEQR
tviv|TvY486_1012140              FIQETLSTMRYTQRLKRIKTKGRPQKPCDDLSLFRPSNQTNLLEELMMLRRIVG------
tcon|TcIL3000.10.10660           FVQETMSTLRYTQRLKRIKTKSAPQKPSDDSSLFHPKEHQDLLEELALLRKIVN------
tbrg|Tbg972.10.14990             FVQETMSTMRYTQRLKHIKTKPPPQKPNDDSSLFHPKEHKNLLEELSALRKIVG------
tbru|Tb927.10.12440              FVQETMSTMRYTQRLKHIKTKPPPQKPNDDSSLFHPKEHKNLLEELLALRKIVG------
                                 :  **:**:******* *.*     .  :  * :.  ::  **:**  **  :

lbra|LbrM18_V2.1630              -----ASENASKLVEAAHQRRI--AELESTL---AKQGRSPCGAVGASLPLLSPPRELNA
lmex|LmxM18.1600                 -----VSENATKLVEATHRRRI--AELEHTL---AKQGGLHDGAAGASAPLSPAPREMNA
linf|LinJ18_V3.1590              -----VSENVTKLVEATHRRRI--AELEHTL---AKQGGSHDGAAGASAPLSSAPREMNA
lmaj|LmjF18.1600                 -----VSENTTKLVEATHRRRI--AELEHTL---AQQGGLHDGAAGASAPLLSAPREVSA
tcru|Tc00.1047053507625.180      EEYHYYNDEFRKVSEETVIGRLPKDHCSDSL---FQNHESALQESSPMISASIVGHRQRL
tviv|TvY486_1012140              ------EKQYASLLRSSKHPGASPDECDGRLDYVSTTANPPQKESAGSSNLGSLNRRRKL
tcon|TcIL3000.10.10660           ---QSSCGEFSPQNDEALAASVCRRHFDPST---PQRETGPETENNDQCNSSGLRHKQRL
tbrg|Tbg972.10.14990             ---INPCAGVAAGNDDTLKTKLYQDNCAPPA---PPQDAAPRVEGPDRSERSIMRHKQRL
tbru|Tb927.10.12440              ---INPCAGVAAGNDDTLKTKLYQDNCAPPA---PPQDAAPRVEGPDRSERSIMRHKQRL
                                                 :                                      .

lbra|LbrM18_V2.1630              SRARDTRRVAGWLLSRVLGELPELNVGYDAYFDAYFPPSVQVIGYVSTMASLVPRIPGD-
lmex|LmxM18.1600                 RRVRDTRRVAGWLLSRVLGDLPELNVGYDAYFDAYFPPSVQVIGYVSAMASLVPRTAGD-
linf|LinJ18_V3.1590              RRARDTRRVAGWLLSRVLGDLPELNVGYDDYFDAYFPPSVQVIGYVSAMASLVPRIASD-
lmaj|LmjF18.1600                 RRARDTRRVAGWLLSRVLGDLPELNVGYDAYFDAYFPPSVQVIGYVSAMASLVPRTAGD-
tcru|Tc00.1047053507625.180      LRYKDAMRVAGWLLSRILSELPELSVGFDDYFDTYLPSQVQVVGYISLMTCMAPRDVNEA
tviv|TvY486_1012140              LRSKDTRRVAGWLLSRILGKLPALSVGFDDYFDPFINDGVQVVGYVSLMACLPPRDAGDA
tcon|TcIL3000.10.10660           IRSKDIRRVAGWLVSRTLGMLPHLSVRFDDYFDDFLPCEVQVVGYVSLMACLPPCDKAEA
tbrg|Tbg972.10.14990             LRSKDIRRVAGWLVSRTLSMLPQLSVGFDDYFDDYLPGEIQVVGYVSLMACLPPCSKTEA
tbru|Tb927.10.12440              LRSKDIRRVAGWLVSRTLSMLPQLSVGFDDYFDDYLPGEIQVVGFVSLMACLPPCSKTEA
                                  * .*  ******:** *. ** *.* :* *** ::   :**:*::* *:.: *    :

lbra|LbrM18_V2.1630              -DPLAFLDVGDLAMGLSMLDAGVPPFVRLHTSRCGDPSGWEGYEWDSSHDVVYVLAFFEY
lmex|LmxM18.1600                 -DPLAFLDVGDLAMGLSMLDAGVPPFVRLHKSVCSDLSNWAGCEWDGTQSVVYVLTFFEY
linf|LinJ18_V3.1590              -DPLAFLDVGDLAMGLSMLDAGVPPLVRLHKSVCSDPSSWEGCEWDGTQNMVYVLAFFEY
lmaj|LmjF18.1600                 -DPLAFLDVGDLAMGLSMLDAGVPPFVRLHKSLCSDPSSWEGCEWDGAQNVVYVLAFFEY
tcru|Tc00.1047053507625.180      MGGLAFLDVGDIAMGLSMLDAGIPACVGLHRLTCGESNTWEAHEYDGVNR-VFVLAFFEI
tviv|TvY486_1012140              TGTLAFLDVGDLVMGFSMLDAGIPACVGLHRLNCGGSSSWVAHEYSEVDK-VFVLAFFEV
tcon|TcIL3000.10.10660           SHSLAFLDVGDPALGLSMLDAGIPACVGLHRLDCKGIRYWEVHEYDETNS-VFLLAFFRV
tbrg|Tbg972.10.14990             LNGLAFLDVGDPSIGLSMLDAGIPACVGLHKLSCKEVHSWEVHEYNETNN-IFLLAFFKV
tbru|Tb927.10.12440              LNGLAFLDVGDPSIGLSMLDAGIPACVGLHKLSCKEVHSWEVHEYNETNN-IFLLAFFKV
                                    ********  :*:******:*. * **   *     *   *:.     :::*:**

lbra|LbrM18_V2.1630              HPTAMSAA------GMEEALQCCGGYVTSAPLLPIATVLCVAESATRRVKEEVLQRLVDL
lmex|LmxM18.1600                 HPTAMSSA-AEDAAGMEEALPCCGGYVTSAPLLPIATVLCVAASTTRRVKEEVLQRLVDL
linf|LinJ18_V3.1590              HPTAMSSATAGDAAGMEEALPCCGGYTTSAPLLPIATVLCVAASTTRRVKEEVLQRLVDL
```

```
lmaj|LmjF18.1600                 HPTAMSPA-AGDAAGMEEALTCCGGYVTSAPLLPIATVLCVAASTTRRVKEEVLQRLVDL
tcru|Tc00.1047053507625.180      NEHLVEIM-----GDTHDAFECCGGLLTLEPLVPLAIVLCTPLDSSDELKENVLQHLVTL
tviv|TvY486_1012140              NEHLVESM-----NGTGEALECCDGLLTLEPLVPLALVLCTPYDASDELKESVLQHLITL
tcon|TcIL3000.10.10660           GEGLVEPM-----DVSGNPFECCGGVLTLEPLVPLALVFGTSRDTCEHVKENVLQHLITL
tbrg|Tbg972.10.14990             DEQLVEPL-----GASGSPFACCGGLLTLEPLMPLALVFGAPLNAPNEVKENVLQHLVTL
tbru|Tb927.10.12440              DEQLVEPL-----GASGSPFACCGGLLTLEPLMPLALVFGAPLNAPNEVKENVLQHLVTL
                                     :.           ..: **.*  *  **:*:* *: .. .:   :**.***.*: *

lbra|LbrM18_V2.1630              QCEQHEAVMEQANAGARTPSLSSSLSSSRQST---LPSGTELLDASLVH--SVGGAEGDN
lmex|LmxM18.1600                 QCEQHEAVAEQAKAGARTPSLSSSMLSSRQDG---FPSDAALLDASEMR--NISEAESDD
linf|LinJ18_V3.1590              QCEQHEAVAEQAKTGARSPSLSSTMLSSRQAG---FPSAASLLDASDMR--NVSEAESDD
lmaj|LmjF18.1600                 QCEQHEAVAEQAKTRARTPSLSSSMLLSRQDG---FPSEASLLDTSDMR--NVSEAESHD
tcru|Tc00.1047053507625.180      QGEQDGAL--ESSIGGSSSLSQLTPMHTGIDGAMENPKLSLLKAMTQWRDDYVKEVTSSS
tviv|TvY486_1012140              QSDQEETS--NCFPVNSASMLQVMSDLHPVDVVVKYPEEEQSKILPDCQ--HGFTQDPDE
tcon|TcIL3000.10.10660           QTEQGHVTEGNAATQTSRSLYEQGSASCENSV---RCGCRALVDTSETT--YSSGVDEET
tbrg|Tbg972.10.14990             QSEQHQPAGASNDTDASQSVHKKSNDSHGSVP---FFNSEVLDALSDYS--YHYEVGEDS
tbru|Tb927.10.12440              QSEQHQPAGASNDTDASQSVHKKSNDSHGSVP---FFNSEVLDALSDYS--YHYEVGEDS
                                 * :*      .       .  .                       .

lbra|LbrM18_V2.1630              DASAISSAANGNAL-LGKEVHNQAADTPCIVAPLKEEDSSTNNSNSHSCDSRKEARAEI-
lmex|LmxM18.1600                 TASASTDSTSGGAF-LAQEVRRQAADTSHIVTPFQEEEECSASGGR-SCDSREEARAEV-
linf|LinJ18_V3.1590              DASASTGGSTSGGAL-LALEARCQAADTPHIATPFQ-EEEGSASGGC-SCDSCEEARAAM-
lmaj|LmjF18.1600                 DASASTGGSTSGGAL-LALELRRQTADTSHIVTPFQEEEEGSASDGR-SCDSREAARAEM-
tcru|Tc00.1047053507625.180      DTESFSAAP------LAEEIERQTLNKLQVCSPFQ-ESASVNSGEAFSNEKTTIIHLDH-
tviv|TvY486_1012140              TTDSIASVMETLKR-EVRHSFRDPLNQSHLSTEKA--QHCETPDIGSLCRNRPFPLVTA-
tcon|TcIL3000.10.10660           EFESIVTIHGTEPPSVLKEGSPQSSTHTPGRSPHSREQSDEVHNPR-RAQFFGGKRSDA-
tbrg|Tbg972.10.14990             LIGSPVG--PQRKA-LTDHNEGQNGDGFRSQISWESVQFQEDLTEMVCCRAVGSADIDEA
tbru|Tb927.10.12440              LIGSPVG--PQRKA-LTDHNEGQNGDGFRSQISWESVQFQEDLTEMVCCRAVGSADIDEA
                                    :                  :              .

lbra|LbrM18_V2.1630              -----------------GSAEV-RHNLGECTAPSRPSVSPPREGKAE-------------
lmex|LmxM18.1600                 -----------------RSAKVHRHLPGELTSPSRSSVPPPRARKAE-------------
linf|LinJ18_V3.1590              -----------------RSAEVHRHLAGELTSPSRPSAPPPRARNAE-------------
lmaj|LmjF18.1600                 -----------------RSAEMHRHLPGELTSLSRPSVPPPRACKAE-------------
tcru|Tc00.1047053507625.180      --KLHAIYASSSSFSSESSSDSSVILGSKLSLLSPAVADEGQCQEIS---------RTHM
tviv|TvY486_1012140              ------PLLSPSSSLSSSFSTAPSTLPQVGANTQEGLTPSPVPRDEAMEQNSYAESNEEQ
tcon|TcIL3000.10.10660           ----SDVLSSLSVSSSNEEGQRDLDVSPGLTEERVACTTSGSTQDQS---------PQST
tbrg|Tbg972.10.14990             DDPEPSSLSSPSTFCGEEKELNPVPLPNSVAVRAVSCDIDPEKGEQS---------PQSV
tbru|Tb927.10.12440              DDPEPSSLSSPSTFCGEEKELNPVPLPNSVAVRAVSCDIDPEKGEQS---------PQSV
                                                               :             .

lbra|LbrM18_V2.1630              ------------------------------------ETKAAEGSTTAAVSFPQSASPT--
lmex|LmxM18.1600                 ------------------------------------EPAQVRGGTAAAVASSQLMPAT--
linf|LinJ18_V3.1590              ------------------------------------ESAKVRGCTAAAVSSPLLMPAT--
lmaj|LmjF18.1600                 ------------------------------------EPSQVRGGSAAAVSSPPLMPAS--
tcru|Tc00.1047053507625.180      SSECPSETECDAEKGVGMPQSIDREVHGANQVGVSVSPADVHVDMYNRPYLPHAPQTN--
tviv|TvY486_1012140              HEGTMDTDTIGVELFQSDSATKSSFVLDHGGEDAARAPCATEVGASVGELPGGTLPAS--
tcon|TcIL3000.10.10660           GEQIITDRTDMCDGNTS-GDPAFRARWLDGNTTLSDEIEQAASNVLPDPVPPPSPQHT--
tbrg|Tbg972.10.14990             GEQIITDSADQCDVETLDDQPSLRAGGDESEISFK-EVELTVGTTANTSSSPGLLESQAK
tbru|Tb927.10.12440              GEQIITDSADQCDVETLDDQPSLHAGGDESEISFK-EVELTVGTTANTSSSPGLLESQPK
                                                                         .

lbra|LbrM18_V2.1630              -------RRLFSPQ------------RPS--------GTALALLPPADEVSPGCTSKRRH
lmex|LmxM18.1600                 -------CKSLSPW------------RTS--------GTSLAPLPPADALPPGHTSKRRH
```

```
linf|LinJ18_V3.1590              -------CKSFSPL------------RPS--------GTSSAPLPPADEFPPGHTSKRRN
lmaj|LmjF18.1600                 -------CKSFSAL------------RTS--------GTSSAPLPPADELPPARTSKRRN
tcru|Tc00.1047053507625.180      -------CYSIEEFPAYAVIARERSKSDKKDDCAECSATASVSPPEKERIYEKNEKKKKK
tviv|TvY486_1012140              -------DQTILPA------------ETVSENTAPHNGPSSVDAPVKQEFEMSTGKKRKN
tcon|TcIL3000.10.10660           -------ASRFTVL------------HDRECTDRTQEGAASDAGAPSGKPSGGKGKNGGD
tbrg|Tbg972.10.14990             SSHILVACEEENRL------------DCPRGEILELSGTLASESPEAHK-GKGKKTKREN
tbru|Tb927.10.12440              SSHILVACEEENRL------------DCPRGEILELSGTLASESPEVHK-GKGKKTKREN
                                                                      ..     .          .:

lbra|LbrM18_V2.1630              R--TAREGKAVKMQTCQGCSAA
lmex|LmxM18.1600                 R--ATGEGKTVHMQSCQGCRSM
linf|LinJ18_V3.1590              R--TAGEGKTVKMQSCQGCRSM
lmaj|LmjF18.1600                 R--IAGEGKTVNMQSCQGCSSM
tcru|Tc00.1047053507625.180      K--NLHRKRKPVLQGCHNCVVL
tviv|TvY486_1012140              R-----RSREPVAHGCHGCTVA
tcon|TcIL3000.10.10660           DGVDSHRKREPVSEGCHLCVML
tbrg|Tbg972.10.14990             R--DNHRDRDPVKQGCHYCVIL
tbru|Tb927.10.12440              R--DNHRDRDPVKQGCHYCVIM
                                         .      *: *
```
