# Supplementary figures and images for: An Essential Farnesylated Kinesin in Trypanosoma brucei
Source: PLoS One. 2011 Nov 2;6(11):e26508. doi: 10.1371/journal.pone.0026508 (PMC3206815; doi:10.1371/journal.pone.0026508)

S3

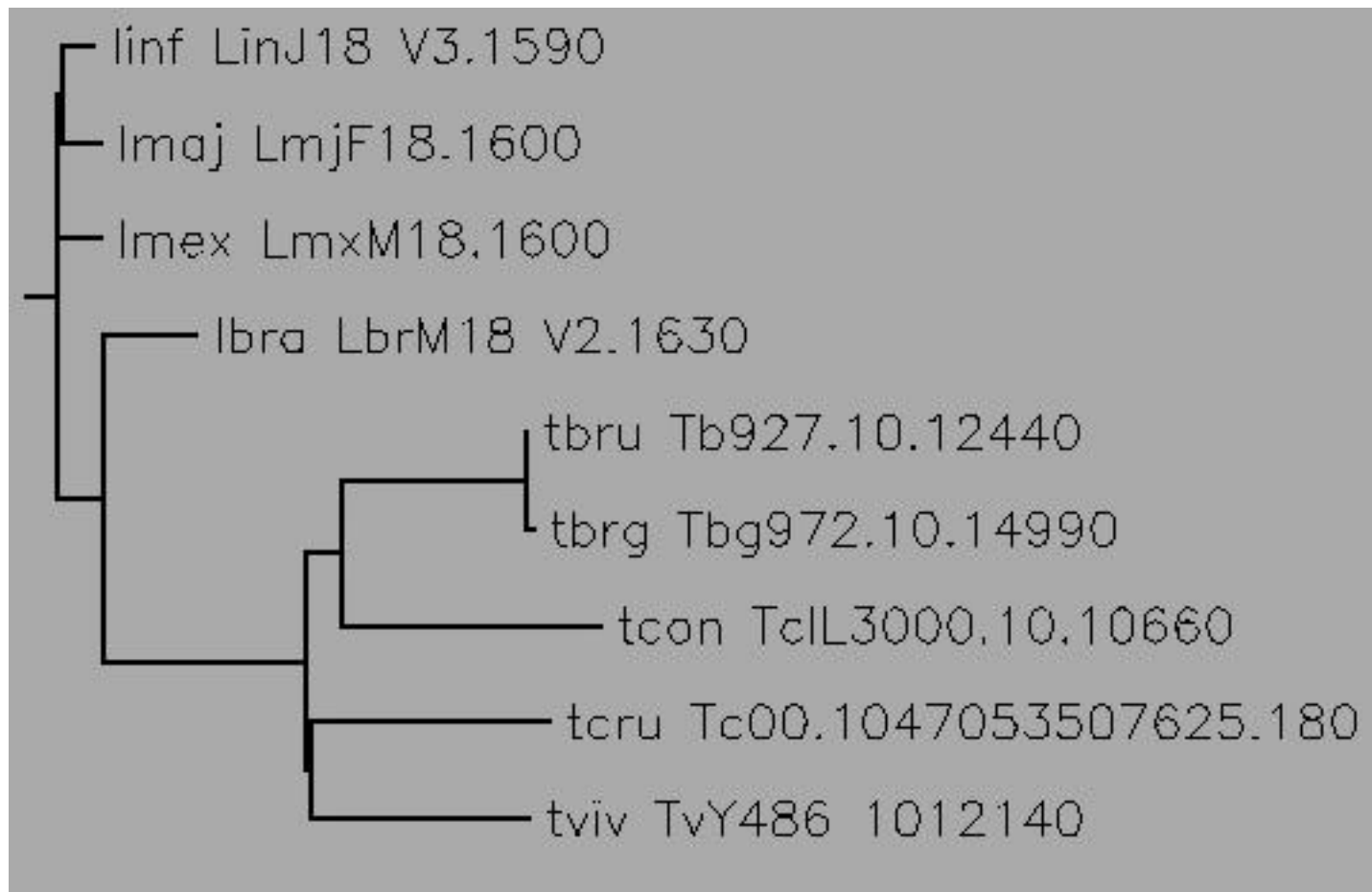

Supplement: Figure S3 — Dendrogram of KinesinCaaX and orthologs from other pathogenic kinetoplastids. The evolutionary relationship of KinesinCaaX was analyzed using Biology Work Bench, Version 3.2, DRAWGRAM and PHYLIP (Phylogeny Inference Package) version 3.5c [26]. This analysis demonstrates the T. brucei gambiense homolog to be most closely related to T. brucei brucei KinesinCaaX followed by the other Trypanosoma species and then the Leishmania species. For gene ID and species key, see Legend of Figure S2. Horizontal distances represent relative genetic distance. (PDF) [file pone.0026508.s003.pdf]

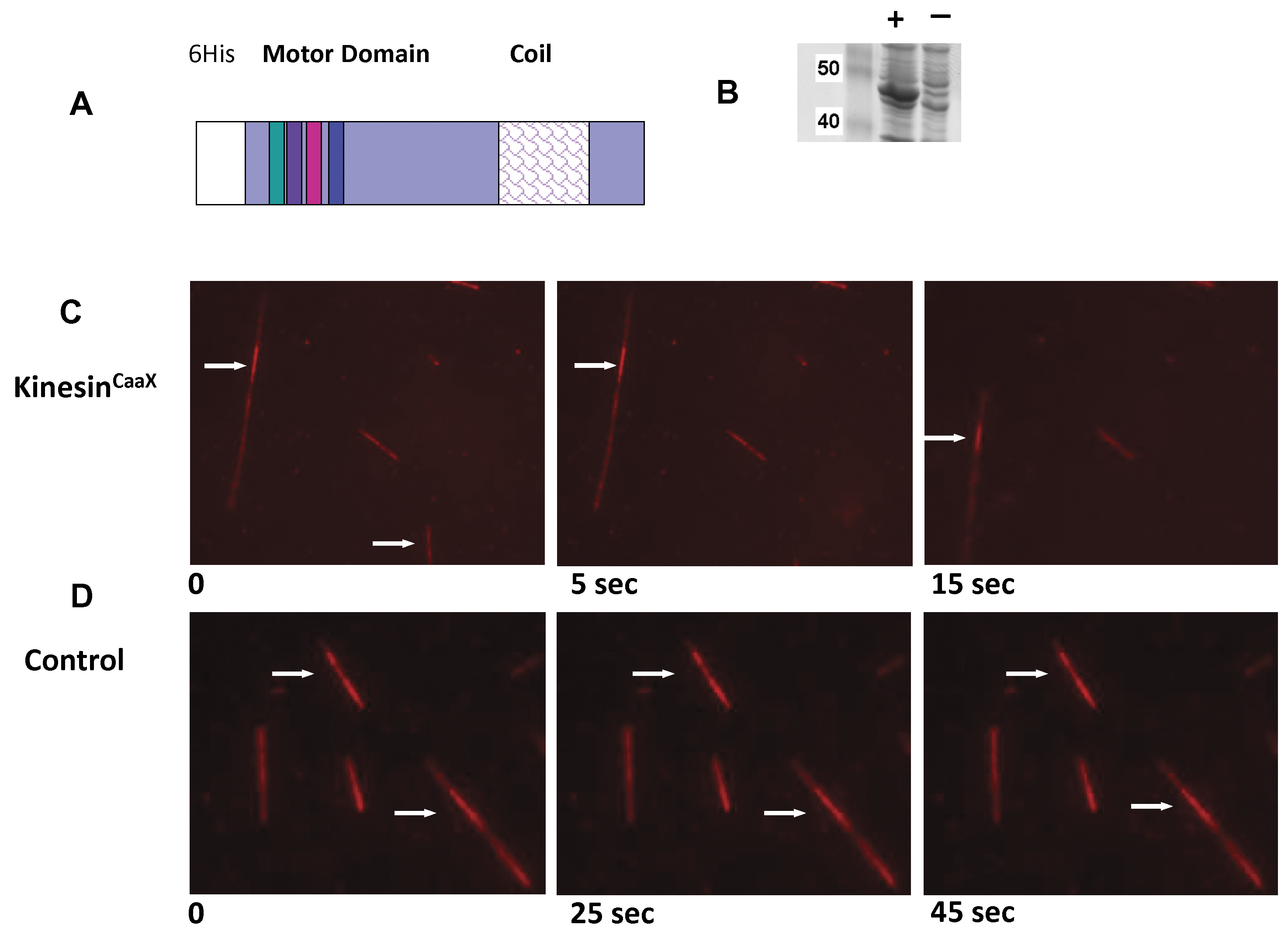

Supplement: Figure S4 — KinesinCaaX has motor activity in the presence of ATP in vitro . (A) Recombinant His-tagged truncated N-terminal 340 AA-construct of KinesinCaaX restricted to the predicted motor domain and the coil region predicted to facilitate dimerization. (B) Coomassie stained PAGE showing E.coli soluble lysate used for the motor assays in (C) with the increased band at the expected molecular weight (44 kDa) for KinesinCaaX motor-domain upon IPTG jnduction (+). (C) An in vitro gliding motility assay using truncated KinesinCaaX and fluorescent microtubules. Lysates containing recombinant truncated KinesinCaaX was allowed to adhere to glass microscope slides. Next rhodamine-labeled microtubules were added with or without ATP to the slide and microtubule motility was monitored by time-lapse fluorescence microscopy. Selected frames from time lapse microscopy are represented. The positions of rhodamine-labeled microtubules over time are marked with arrows. (D) Control cell lysates containing recombinant β-galactosidase non-motor protein control showed no microtubule movement in the presence of ATP. The positions of two stationary microtubules are marked with arrows. (TIF) [file pone.0026508.s004.tif]

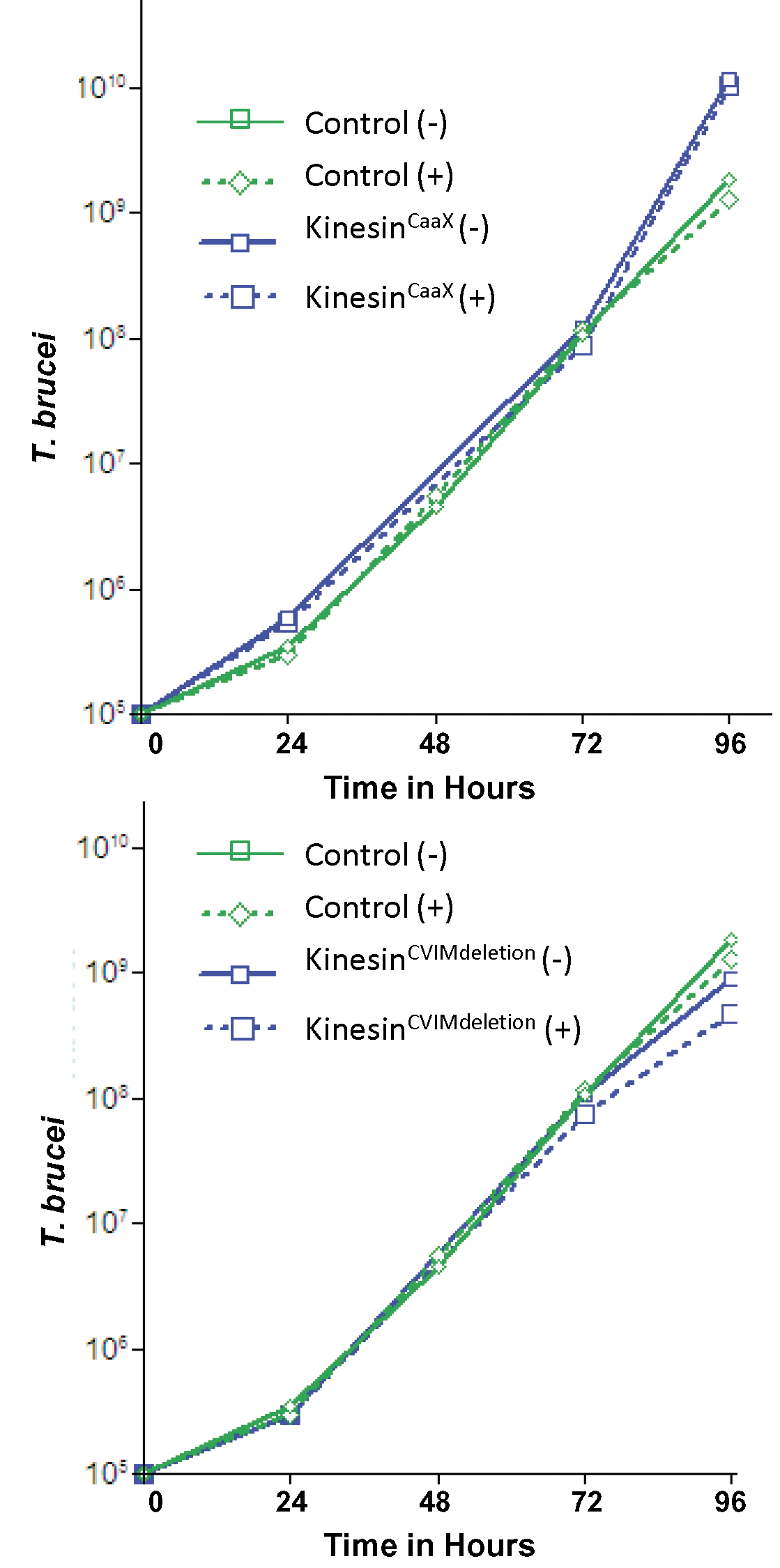

Supplement: Figure S5 — Growth rate of T. brucei exogenously expressing KinesinCaaX is not affected after induction but KinesinCVIMdeletion induced cells begin to slow their growth rate at 96 hrs. Shown are growth curves of blood-stream T. brucei parasites grown either with (+) or without (−) tetracycline induction. Control parasites are “Single Marker” parasites without an exogenous expression plasmid. KinesinCaaX and KinesinCVIMdeletion are parasites transfected with the exogenous expression vector expressing these proteins under control of tetracycline induction. Parasite numbers are shown on the Y axis and time after tetracycline induction in hours is shown on the X axis. The experiment shown represents a single clone from each transfection but the same results were also observed with a second clone from each transfection (data not shown). (TIF) [file pone.0026508.s005.tif]
